# Supplementary material for: Effectiveness of Local Antibiotics for Infection Prevention in Primary Joint Arthroplasty: A Systematic Review and Meta-Analysis
Source: Antibiotics (Basel). 2025 Feb 20;14(3):214. doi: 10.3390/antibiotics14030214 (PMC11939600; doi:10.3390/antibiotics14030214)
Supplement: Supplementary file 1 [file antibiotics-14-00214-s001.zip › Supplementary Material File S1.pdf]

Supplementary Material File S1

Effectiveness of Local Antibiotics for Infection Prevention in Primary Joint Arthroplasty: A Systematic Review and Meta-Analysis

Content

Search strategy

Table S1. Characteristics of The Included Studies

Table S2. Risk of Bias for Cohort Studies Evaluating Deep Wound Infection or PJI Using the NOS

Table S3. The Dosage and Administration Timing of Local Antibiotics of the Included Studies

Table S4. Isolated Gram-positive Aerobic Pathogens from All Local Antibiotics

Table S5. Isolated Gram-positive Aerobic Pathogens from Local Vancomycin Powder

Figure S1. Risk of Bias for RCTs Evaluating Deep Wound Infection or PJI

Figure S2. Funnel Plot Asymmetry Results of Publication Bias

Figure S3. Sensitivity Analysis of RCT Subgroup Excluding Abuzaiter 2023 [7] and Hinarejos 2013 [16]

Figure S4. Sensitivity Analysis of Cohort Study Subgroup Excluding Namba 2009 [37]

Figure S5. Forest Plot of RCTs without Subgrouping by Administration Type

Figure S6. Sensitivity Analysis of RCT without Subgroup Excluding Hinarejos 2013 [16]

Figure S7. Forest Plot of Cohort Studies without Subgrouping by Administration Type

Figure S8. Sensitivity Analysis of Cohort Study without Subgroup Excluding Namba 2009 [37]

Figure S9. Forest Plot of Combined RCTs and Cohort Studies without Subgrouping by Administration Type

Figure S10. Sensitivity Analysis of RCT and Cohort Study without Subgroup Excluding Namba 2009 [37] and Hinarejos 2013 [16]

Figure S11. Forest Plot of Combined RCTs and Cohort Studies with Subgroup

Figure S12. Sensitivity Analysis of RCT and Cohort Study with Subgroup Excluding Namba 2009 [37] and Hinarejos 2013 [16]

Figure S13. Funnel Plot of Powder Administration Data

Figure S14. Forest Plot of RCTs Using Vancomycin Powder with Dosage Subgroup

Figure S15. Forest Plot of Cohort Studies Using Vancomycin Powder with Dosage Subgroup

Figure S16. Sensitivity Analysis of RCT and Cohort Study with Dosage Subgroup Excluding Abuzaiter 2023 [7]

1. Search strategy (access date September 7, 2024)

Search strategy for PubMed

1. arthroplasty (128,948)
2. cementoplasty (3920)
3. joint prosthesis (114,518)
4. local (1,980,378)
5. topical (150,620)
6. anti-bacterial agents (876,923)
7. antibiotics (1,096,229)
8. surgical (4,512,732)
9. prophylaxis (1,804,315)
10. prophylactic (119,134)
11. 1 or 2 or 3 (154,680)
12. 4 or 5 (2,107,830)
13. 6 or 7 (1,112,018)
14. 8 or 9 or 10 (6,050,681)
15. 11 and 12 and 13 and 14 (749)

Search strategy for EMBASE

1. cementoplasty/exp or cementoplasty or arthroplasty/exp or alloarthroplasty or arthroplasties or arthroplasty or joint reconstruction or joint reconstructive procedure or joint reconstructive surgery (168,379)

2. local OR topical (1,507,336)
3. surgical or prophylaxis/exp or prophylactic (3,862,354)
4. antibiotic agent/exp or antibiotic or antibiotic agent or antibiotic drug or antibiotic ointment or antibiotics (2,293,312)
5. 1 and 2 and 3 and 4 (710)

Search strategy for Cochrane library

1. MeSH descriptor: [Arthroplasty] explode all trees (8111)
2. MeSH descriptor: [Cementoplasty] explode all trees (280)
3. MeSH descriptor: [Anti-Bacterial Agents] explode all trees (17,055)
4. arthroplasty (17,037)
5. cementoplasty (18)
6. anti-bacterial agents (15,506)
7. local (100,227)
8. topical (39,166)
9. surgical (146,419)
10. prophylaxis (31,952)
11. prophylactic (20,987)
12. antibiotics (23,706)
13. 1 or 2 or 4 or 5 (18,165)
14. 3 or 6 or 12 (34,837)
15. 7 or 8 (132,750)

16. 9 or 10 or 11 (185,209)

17. 13 and 14 and 15 and 16 (62)

2. Supplementary Tables

Table S1. Characteristics of the included studies

Author	Design	Patients (control/treatment)	Age (average)	Local antibiotics	Administration	Follow-up period	Primary outcome	Infection rate
Abuzaiter 2023 [7]	RCT	165 (85/80)	control: 64; treatment: 66	Vancomycin	Powder	42 days (at least 1 month)	Deep infection	control: 0/85 (0%); treatment: 3/80 (3.8%)
Aljuhani 2021 [24]	Cohort	98 (49/49)	NR	Vancomycin	Powder	3 months	Deep infection	control: 1/49 (2.0%); treatment: 0/49 (0%)
Assor 2010 [25]	Cohort	135 (73/62)*	control: 72; treatment: 73	Vancomycin	Powder	at least 3 years	Deep infection	control: 3/73 (4.1%); treatment: 0/62 (0%)
Buchalter 2021 [26]	Cohort	18,299 (3982/14,317)*	NR	Vancomycin	Powder	3 months	Deep infection	control: 32/3982 (0.8%); treatment: 71/14,317 (0.5%)
Buchalter 2021 (2) [27]	Cohort	9228 (2182/7046)*, †	NR	Vancomycin	Powder	3 months	Deep infection	control: 22/2182 (1.0%); treatment: 31/7046 (0.4%)
Chin 2018 [13]	RCT	22 (11/11)	control: 63; treatment: 66	Vancomycin	Intraosseous regional administration	6 months	Deep infection	control: 0/11 (0%); treatment: 0/11 (0%)
Chiu 2001 [14]	RCT	78 (37/41)*	control: 69; treatment: 72	Cefuroxime	Antibiotic-impregnated cement	at least 2 years	Deep infection	control: 5/37 (13.5%); treatment: 0/41 (0%)
Chiu 2002 [15]	RCT	340 (162/178)*	control: 68; treatment: 70	Cefuroxime	Antibiotic-impregnated cement	at least 2 years	Deep infection	control: 5/162 (3.1%); treatment: 0/178 (0%)
Cohen 2019 [28]	Cohort	555 (246/309)	control: 67.3; treatment: 66	Vancomycin	Powder	NR	Periprosthetic joint infection	control: 4/246 (1.6%); treatment: 2/309 (0.7%)
Crawford 2018 [29]	Cohort	1885 (815/1070)*	control: 63.3; treatment: 64.8	Vancomycin	Powder	NR	Deep and overall infection	(deep) control: 7/815 (0.9%); treatment: 1/1070 (0.1%) (overall) control: 12/815 (1.5%); treatment: 5/1070 (0.5%)

Dial 2018 [30]	Cohort	265 (128/137)	control: 61.5; treatment: 61.2	Vancomycin	Powder	3 months	Deep infection	control: 7/128 (5.5%); treatment: 1/137 (0.7%)
Erken 2020 [31]	Cohort	93 (58/35)	control: 81.9; treatment: 81.9	Vancomycin	Powder	NR	Overall infection	control: 4/58 (6.9%); treatment: 2/35 (5.7%)
Hanada 2019 [32]	Cohort	202 (92/110)*	control: 73.3; treatment: 74.6	Vancomycin	Powder	at least 1 year	Periprosthetic joint infection	control: 7/92 (7.6%); treatment: 5/110 (4.6%)
Harper 2020 [33]	Cohort	200 (100/100) ¶	control: 67; treatment: 67	Vancomycin	Intraosseous regional administration	3 months	Deep infection	control: 0/100 (0%); treatment: 0/100 (0%)
Hinarejos 2013 [16]	RCT	2948 (1465/1483)*	control: 76.1; treatment: 75.8	Erythromycin and Colistin	Antibiotic-impregnated cement	at least 1 year	Deep and overall infection	(deep) control: 20/1465 (1.4%); treatment: 20/1483 (1.4%); (overall) control: 38/1465 (23.1%); treatment: 47/1483 (3.2%)
Josefsson 1993 [17]	RCT	1688 (835/853)*	68 §	Gentamicin	Antibiotic-impregnated cement	at least 1-2 years	Deep infection	control: 13/835 (1.6%); treatment: 3/853 (0.4%)
Khatri 2017 [34]	Cohort	115 (64/51)	NR	Vancomycin	Powder	6 months	Deep and overall infection	(deep) control: 6/64 (9.4%); treatment: 4/51 (7.8%); (overall) control: 8/64 (12.5%); treatment: 5/51 (9.8%)
Klasan 2021 [35]	Cohort	631 (331/301)*	control: 68.7; treatment: 67.7	Vancomycin	Intraosseous regional administration	3 months and 1 year	Periprosthetic joint infection	(3 months) control: 0/331 (0%); treatment: 0/301 (0%); (1 year) control: 0/331 (0%); treatment: 1/301 (0.3%)
Koutalos 2020 [36]	Cohort	TKA: 176 (93/83); THA: 144 (85/59)‡	(TKA) control: 70.5; treatment: 70.3; (THA) control: 62.5; treatment: 62.1	Vancomycin	Powder	2 years	Deep infection	(TKA) control: 1/93 (1.1%); treatment: 1/83 (1.2%); (THA) control: 1/85 (1.2%); treatment: 1/59 (1.7%)
Matziolis 2020 [8]	Cohort	8945 (7863/1082)*	control: 68; treatment: 69	Vancomycin	Powder	1 year	Periprosthetic joint infection	control: 92/7863 (1.2%); treatment: 4/1082 (0.4%)

McQueen 1990 [18]	RCT	405 (201/204)	control: 67; treatment: 67	Cefuroxime	Antibiotic-impregnated cement	2 years	Deep infection	control: 2/201 (1%); treatment: 2/204 (1%)
Mulpur 2024 [19]	RCT	1022 (515/507)	control: 61.4; treatment: 61.7	Vancomycin	Powder	1 year	PJI and overall infection	(PJI) control: 3/515 (0.6%); treatment: 1/507 (0.2%); (overall) control: 2/515 (0.4%); treatment: 1/507 (0.2%)
Namba 2009 [37]	Cohort	22,889 (20,859/2030)	control: 68.1; treatment: 67.5	NR	Antibiotic-impregnated cement	1 year	Deep infection	control: 154/20,859 (0.7%); treatment: 28/2030 (1.4%)
Park 2021 [38]	Cohort	1060 (572/488)	control: 66.7; treatment: 67.4	Vancomycin	Intraosseous regional administration	1 month	Periprosthetic joint infection	control: 3/572 (0.52%); treatment: 0/488 (0%)
Parkinson 2021 [39]	Cohort	1906 (1181/725)*	control: 67; treatment: 67	Cefazolin or Vancomycin	Intraosseous regional administration	at least 1 year	Periprosthetic joint infection	control: 16/1181 (1.4%); treatment: 1/725 (0.1%)
Patel 2018 [40]	Cohort	460 (112/348)*	control: 64.9; treatment: 63.6	Vancomycin	Powder	3 months	Deep and overall infection	(deep) control: 3/112 (2.7%); treatment: 1/348 (0.3%); (overall) control: 3/112 (2.7%); treatment: 2/348 (0.6%)
Tahmasebi 2021 [41]	Cohort	2024 (314/1710)	control: 66.4; treatment: 65	Vancomycin	Powder	at least 1 year	Periprosthetic joint infection	control: 6/314 (1.9%); treatment: 7/1710 (0.4%)
Wang 2023 [20]	RCT	90 (45/45)	control: 68; treatment: 67.9	Vancomycin	Powder	3 months	Periprosthetic joint infection	control: 6/45 (13.3%); treatment: 0/45 (0%)
Wininger 2024 [21]	RCT	20 (10/10)	control: 67; treatment: 69	Vancomycin	Intraosseous regional administration	3 months	Deep infection	control: 0/10 (0%); treatment: 0/10 (0%)
Wu 2022 [22]	RCT	90 (45/45)	control: 68; treatment: 67.9	Vancomycin	Powder	3 months	Periprosthetic joint infection	control: 4/45 (8.9%); treatment: 0/45 (0%)
Xu 2020 [42]	Cohort	855 (418/437)	control: 67.1; treatment: 66.9	Vancomycin	Powder	1.5 years	Periprosthetic joint infection	control: 5/418 (1.2%); treatment: 0/437 (0%)

Yavuz 2020 [43]	Cohort	976 (502/474)	control: 63.4; treatment: 65.5	Vancomycin	Powder	2 year	Periprosthetic joint infection	control: 5/502 (1.0%); treatment: 4/474 (0.8%)
Zhengyuan 2024 [23]	RCT	120 (60/60)	control: 66.5; treatment: 68.3	Vancomycin	Powder	1 year	Periprosthetic joint infection	control: 0/60 (0%); treatment: 0/60 (0%)

Abbreviations: RCT, randomized controlled trial; NR, not reported; TKA, total knee arthroplasty; THA, total hip arthroplasty; PJI, periprosthetic joint infection.

* denotes number of procedures. † high-risk group data excluded. ¶ revision data excluded. § mean data combined with two groups. ‡ group data with tranexamic acid group excluded.

Table S2. Risk of Bias for Cohort Studies Evaluating Deep Wound Infection or PJI Using the NOS

Author (Year)	Risk of bias			
	**Selection**	**Comparability**	**Outcome**	**Overall**
Aljuhani 2021 [24]	****	**	***	9
Assor 2010 [25]	****	**	***	9
Buchalter 2021 [26]	****	**	***	9
Buchalter 2021 (2) [27]	****	**	***	9
Cohen 2019 [28]	****	**	*	7
Crawford 2018 [29]	****	*	*	6
Dial 2018 [30]	****	**	***	9
Erken 2020 [31]	****	*	**	7
Hanada 2019 [32]	****	**	***	9
Harper 2020 [33]	****	**	***	9
Khatri 2017 [34]	****	**	***	9
Klasan 2021 [35]	****	**	***	9
Koutalos 2020 [36]	****	*	***	8
Matziolis 2020 [8]	****	**	***	9
Namba 2009 [37]	****	**	***	9
Park 2021 [38]	****	**	**	8
Parkinson 2021 [39]	****	**	***	9
Patel 2018 [40]	****	**	***	9
Tahmasebi 2021 [41]	****	**	***	9
Xu 2020 [42]	****	**	***	9
Yavuz 2020 [43]	****	**	***	9

One star is equivalent to one point. The highest NOS score is 9.

Abbreviations: PJI, periprosthetic joint infection; NOS, Newcastle-Ottawa Scale

Table S3. The Dosage and Administration Timing of Local Antibiotics of the Included Studies

Author	Local antibiotics	Administration	Dosage	Administration timing of local antibiotics
Abuzaiter 2023 [7]	Vancomycin	Powder	1 g	0.5 g directly around the prosthesis and 0.5 g above the closed joint capsule before wound closure
Aljuhani 2021 [24]	Vancomycin	Powder	2 g	Applied to the joint prior to closure of the fascia
Assor 2010 [25]	Vancomycin	Powder	1-2 g	Vancomycin powder with the paste was spread into a thin layer before setting the implants
Buchalter 2021 [26]	Vancomycin	Powder	2 g	1 g of powder is placed deep to the fascia and 1g is placed superficial to the fascia
Buchalter 2021 (2) [27]	Vancomycin	Powder	2 g	1 g of powder is placed deep to the fascia and 1g is placed superficial to the fascia
Chin 2018 [13]	Vancomycin	Intraosseous regional administration	0.5 g	Administered as a bolus immediately after tourniquet inflation, and surgical incision occurred immediately (<1 minute) after this
Chiu 2001 [14]	Cefuroxime	Antibiotic-impregnated cement	2 g	Cement was used to fix the patellar and tibial components
Chiu 2002 [15]	Cefuroxime	Antibiotic-impregnated cement	2 g	Cement was used to fix the patellar and tibial components
Cohen 2019 [28]	Vancomycin	Powder	1 g	Both acetabular and femoral components were dipped in sterile saline, coated with a thin layer of topical vancomycin powder by hand, and then implanted
Crawford 2018 [29]	Vancomycin	Powder	1 g	Sprinkled in the wound after component implantation and prior to closure
Dial 2018 [30]	Vancomycin	Powder	1 g	Applied both intracapsularly and extracapsularly into the wound after final wound irrigation
Erken 2020 [31]	Vancomycin	Powder	1 g	In the surgical wound just before surgical closure
Hanada 2019 [32]	Vancomycin	Powder	1 g	Intracapsularly after final intrawound lavage with saline solution and was not administered extracapsularly if possible
Harper 2020 [33]	Vancomycin	Intraosseous regional administration	0.5 g (500 mg vancomycin in 200 mL normal saline)	100 mL of the vancomycin solution administered via syringe. The device was then removed and inserted into the anterior distal femur, centrally just proximal to the patella for administration of the remaining 100 mL of the mixed solution
Hinarejos 2013 [16]	Erythromycin and Colistin	Antibiotic-impregnated cement	0.5 g of erythromycin and three million units of colistin in 40 g of cement	The cement was mechanically mixed under vacuum conditions

Josefsson 1993 [17]	Gentamicin	Antibiotic-impregnated cement	NR	NR
Khatri 2017 [34]	Vancomycin	Powder	1 g	Applied in subfascial layer just before wound closure
Klasan 2021 [35]	Vancomycin	Intraosseous regional administration	0.5 g	Powder diluted in 150 mL of saline, placed in the proximal tibia, just medial to the tibial tubercle
Koutalos 2020 [36]	Vancomycin	Powder	2 g	After insertion of all the implants and before wound closure, vancomycin powder were placed into the joint space
Matziolis 2020 [8]	Vancomycin	Powder	1 g	Applied intraarticularly before wound closure
McQueen 1990 [18]	Cefuroxime	Antibiotic-impregnated cement	1.5 g	Cefuroxime powder was mixed with cement powder. The liquid polymer was added and the operation continued in the usual way
Mulpur 2024 [19]	Vancomycin	Powder	2 g	Intra-articular antibiotic powder before arthrotomy closure
Namba 2009 [37]	NR	Antibiotic-impregnated cement	NR	NR
Park 2021 [38]	Vancomycin	Intraosseous regional administration	0.5 g (500 mg dose of vancomycin mixed in 150 mL of normal saline)	Injected into proximal tibia after tourniquet inflation before skin incision
Parkinson 2021 [39]	Cefazolin or Vancomycin	Intraosseous regional administration	1 g cefazolin or 500 mg vancomycin was added to 100 mL to 200 mL of saline	Injected immediately before making the skin incision for the procedure
Patel 2018 [40]	Vancomycin	Powder	1 g	Surgical wound before closure in primary hip and knee arthroplasties; applied on the joint and surrounding muscle, fascia, and subcutaneous tissues
Tahmasebi 2021 [41]	Vancomycin	Powder	1 g	At the end of TKA surgeries right before tightly closing the joint capsule
Wang 2023 [20]	Vancomycin	Powder	1-2 g	Vancomycin (0.5 g) was injected into the acetabular fossa at the surgical incision. Following the femoral head mold test, the joint was dislocated, and another dose of vancomycin (0.5 g) was injected into the medullary cavity; For patients with unilateral joint replacement, 1 g vancomycin powder was injected at incision site. At the same time, one gram of vancomycin powder was injected on both sides of the surgical wound of patients undergoing bilateral joint replacement

Wininger 2024 [21]	Vancomycin	Intraosseous regional administration	0.5 g	Delivered via the IO technique at the time of the incision
Wu 2022 [22]	Vancomycin	Powder	1 g	0.5 g of vancomycin powder was placed into the acetabular fossa, and 0.5 g of vancomycin powder injection was placed into the medullary cavity
Xu 2020 [42]	Vancomycin	Powder	0.5 g	Sprinkled evenly onto the surface of synovium, bony elements, muscle and fascia
Yavuz 2020 [43]	Vancomycin	Powder	2 g	Applied to the joint just before the fascia was closed
Zhengyuan 2024 [23]	Vancomycin	Powder	1 g	Applied to the joint just before the fascia was closed

Abbreviations: NR: not reported; TKA: total knee arthroplasty; IO: intraosseous

Table S4. Isolated Gram-positive Aerobic Pathogens from All Local Antibiotics

Pathogens	Control (n)	Intervention (n)
MRSA	12	8
MSSA	56	44
**Other gram-positive aerobic pathogens**		
CoNS	66	13
Streptococcus	15	9
Enterococcae	6	0

Abbreviations: MRSA: methicillin-resistant *Staphylococcus aureus*; MSSA: Methicillin-sensitive *Staphylococcus aureus*; CoNS: Coagulase-negative *Staphylococcus*

Table S5. Isolated Gram-positive Aerobic Pathogens from Local Vancomycin Powder

Pathogens	Control (n)	Intervention (n)
MRSA	11	8
MSSA	40	38
**Other gram-positive aerobic pathogens**		
CoNS	53	12

Streptococcus	13	7
Enterococcae	6	0

Abbreviations: MRSA: methicillin-resistant *Staphylococcus aureus*; MSSA: Methicillin-sensitive *Staphylococcus aureus*; CoNS: Coagulase-negative *Staphylococcus*

3. Supplementary Figures

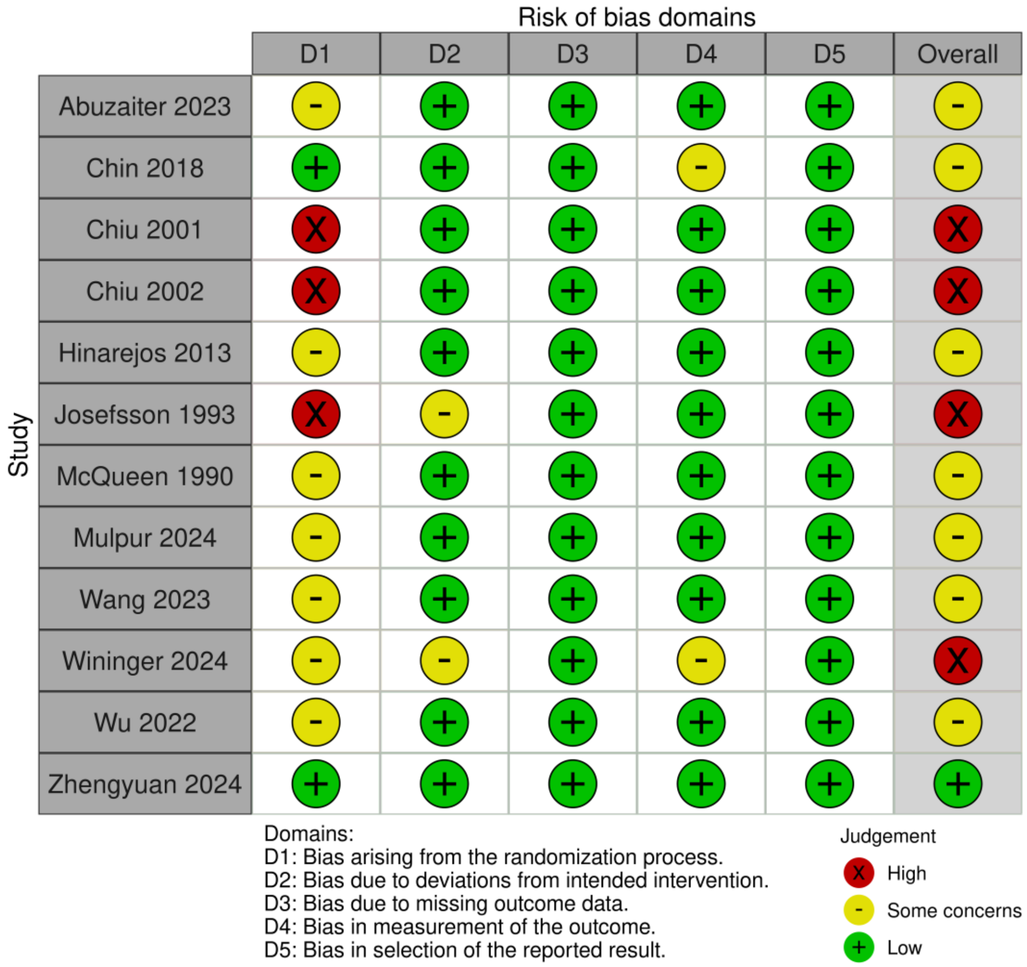

Figure S1. Risk of Bias for RCTs Evaluating Deep Wound Infection or PJI

Abbreviations: RCTs, randomized controlled trials; PJI, periprosthetic joint infection

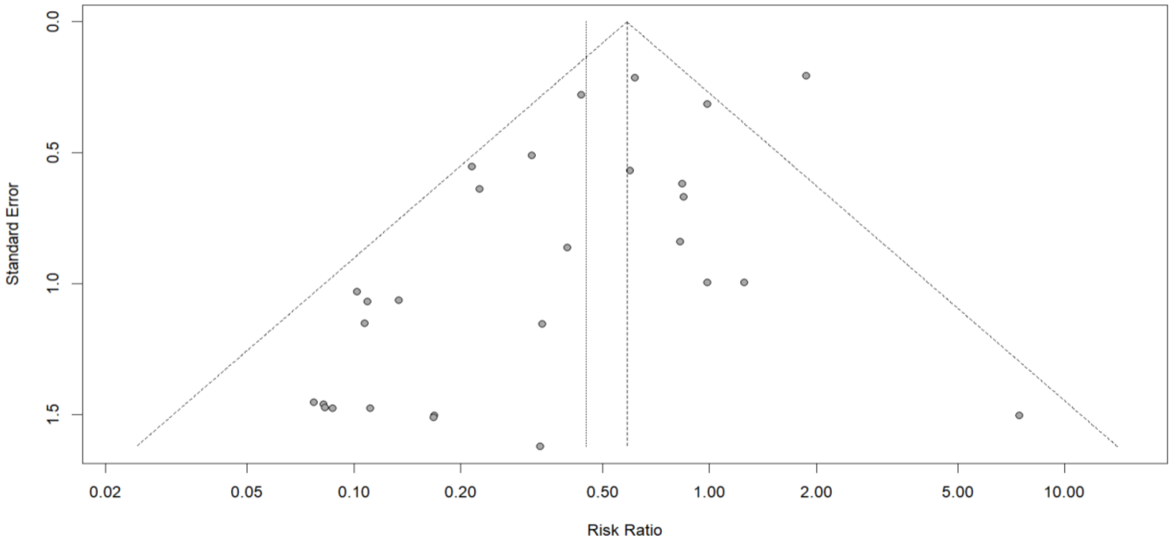

Figure S2. Funnel Plot Asymmetry Results of Publication Bias (Peters' test: p = 0.1415; Egger's test: p =

0.0014)

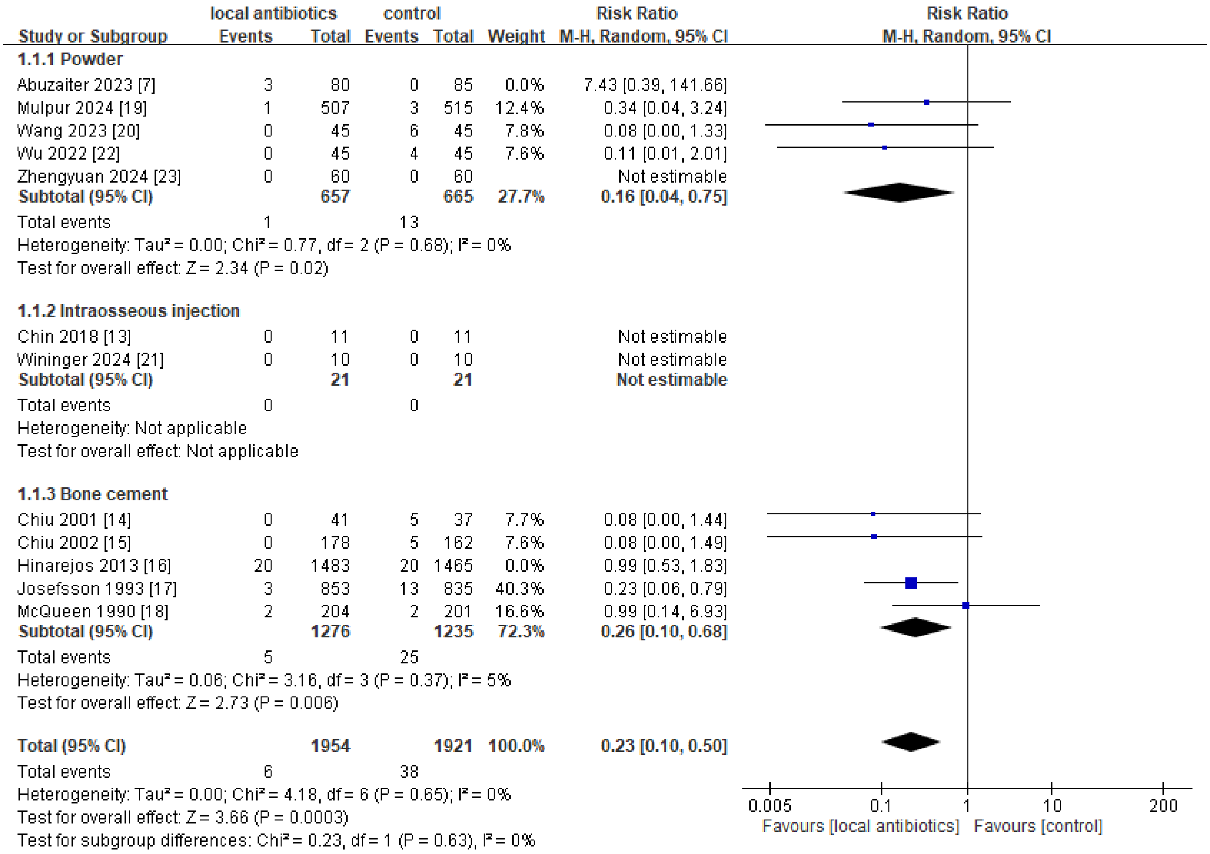

Figure S3. Sensitivity Analysis of RCT Subgroup Excluding Abuzaiter 2023 [7] and Hinarejos 2013 [16]

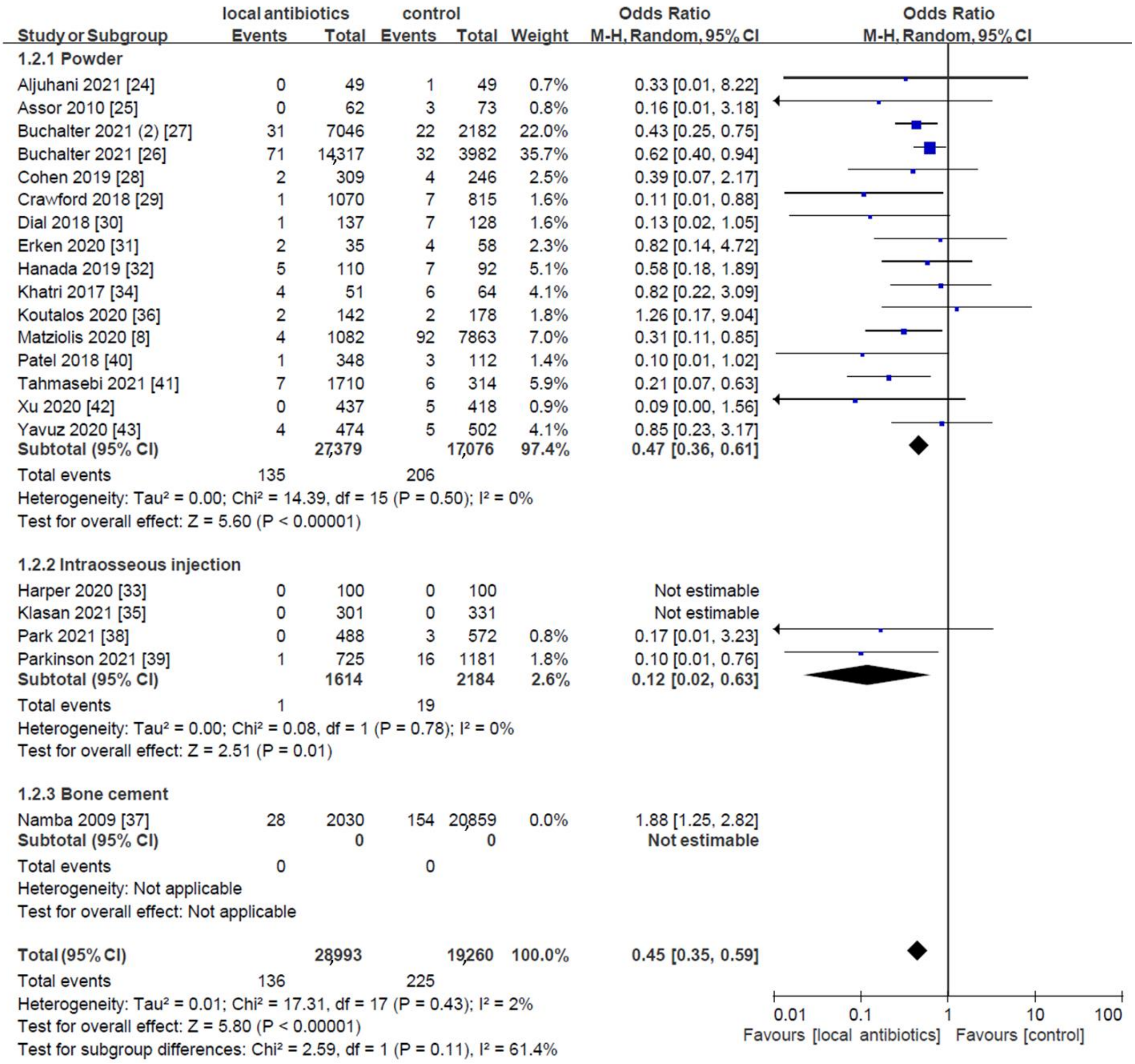

Figure S4. Sensitivity Analysis of Cohort Study Subgroup Excluding Namba 2009 [37]

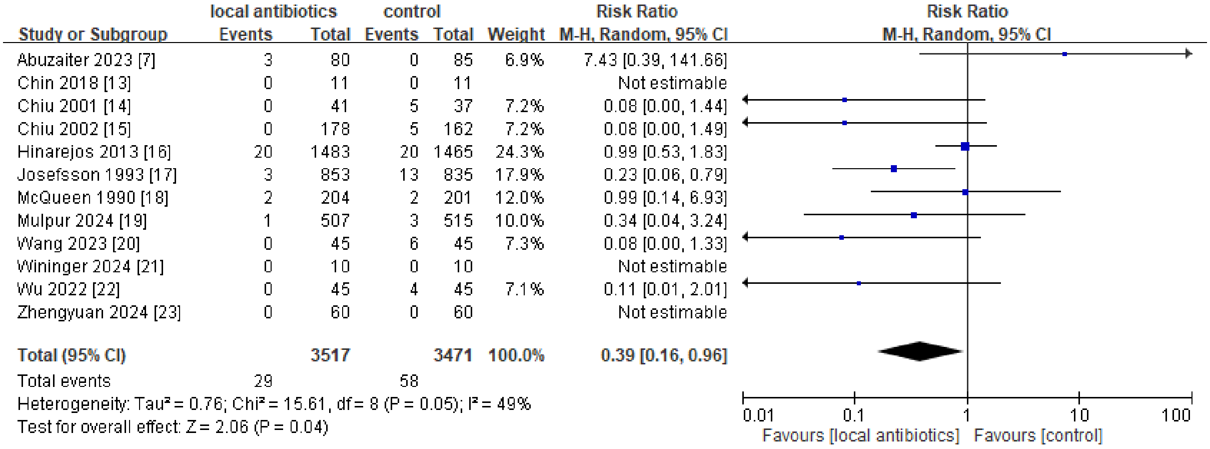

Figure S5. Forest Plot of RCTs without Subgrouping by Administration Type

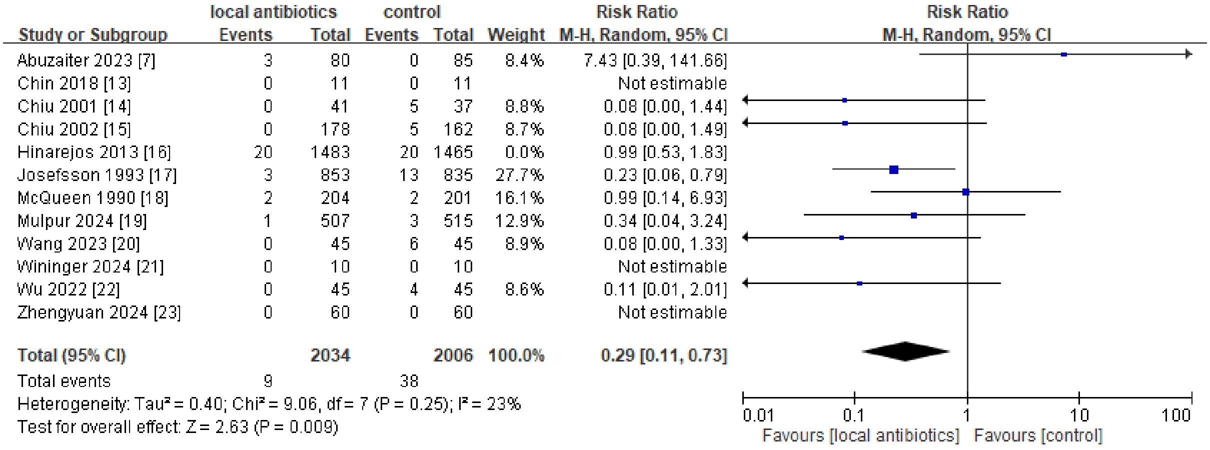

Study or Subgroup	local antibiotics Events	local antibiotics Total	control Events	control Total	Weight	Risk Ratio M-H, Random, 95% CI
Abuzaiter 2023 [7]	3	80	0	85	8.4%	7.43 [0.39, 141.66]
Chin 2018 [13]	0	11	0	11		Not estimable
Chiu 2001 [14]	0	41	5	37	8.8%	0.08 [0.00, 1.44]
Chiu 2002 [15]	0	178	5	162	8.7%	0.08 [0.00, 1.49]
Hinarejos 2013 [16]	20	1483	20	1465	0.0%	0.99 [0.53, 1.83]
Josefsson 1993 [17]	3	853	13	835	27.7%	0.23 [0.06, 0.79]
McQueen 1990 [18]	2	204	2	201	16.1%	0.99 [0.14, 6.93]
Mulpur 2024 [19]	1	507	3	515	12.9%	0.34 [0.04, 3.24]
Wang 2023 [20]	0	45	6	45	8.9%	0.08 [0.00, 1.33]
Wininger 2024 [21]	0	10	0	10		Not estimable
Wu 2022 [22]	0	45	4	45	8.6%	0.11 [0.01, 2.01]
Zhengyuan 2024 [23]	0	60	0	60		Not estimable
**Total (95% CI)**		**2034**		**2006**	**100.0%**	**0.29 [0.11, 0.73]**
Total events	9		38			

Heterogeneity: Tau² = 0.40; Chi² = 9.06, df = 7 (P = 0.25); I² = 23%
Test for overall effect: Z = 2.63 (P = 0.009)

Figure S6. Sensitivity Analysis of RCT without Subgroup Excluding Hinarejos 2013 [16]

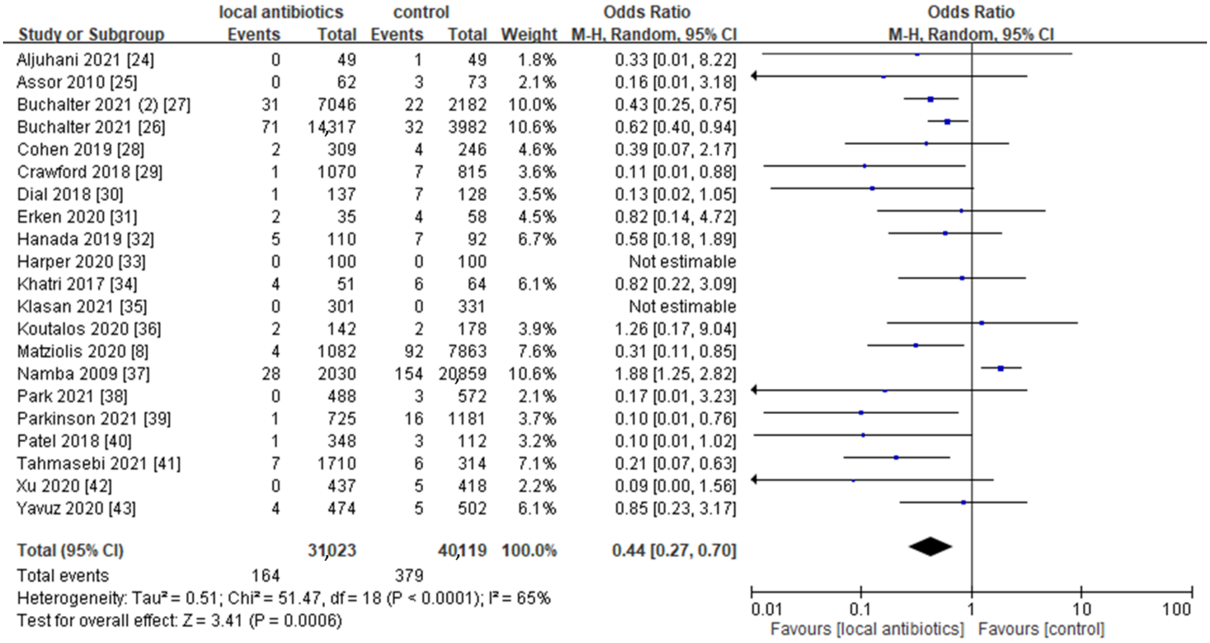

Study or Subgroup	local antibiotics Events	local antibiotics Total	control Events	control Total	Weight	Odds Ratio M-H, Random, 95% CI
Aljuhani 2021 [24]	0	49	1	49	1.8%	0.33 [0.01, 8.22]
Assor 2010 [25]	0	62	3	73	2.1%	0.16 [0.01, 3.18]
Buchalter 2021 (2) [27]	31	7046	22	2182	10.0%	0.43 [0.25, 0.75]
Buchalter 2021 [26]	71	14,317	32	3982	10.6%	0.62 [0.40, 0.94]
Cohen 2019 [28]	2	309	4	246	4.6%	0.39 [0.07, 2.17]
Crawford 2018 [29]	1	1070	7	815	3.6%	0.11 [0.01, 0.88]
Dial 2018 [30]	1	137	7	128	3.5%	0.13 [0.02, 1.05]
Erken 2020 [31]	2	35	4	58	4.5%	0.82 [0.14, 4.72]
Hanada 2019 [32]	5	110	7	92	6.7%	0.58 [0.18, 1.89]
Harper 2020 [33]	0	100	0	100		Not estimable
Khatri 2017 [34]	4	51	6	64	6.1%	0.82 [0.22, 3.09]
Klasan 2021 [35]	0	301	0	331		Not estimable
Koutalos 2020 [36]	2	142	2	178	3.9%	1.26 [0.17, 9.04]
Matziolis 2020 [8]	4	1082	92	7863	7.6%	0.31 [0.11, 0.85]
Namba 2009 [37]	28	2030	154	20,859	10.6%	1.88 [1.25, 2.82]
Park 2021 [38]	0	488	3	572	2.1%	0.17 [0.01, 3.23]
Parkinson 2021 [39]	1	725	16	1181	3.7%	0.10 [0.01, 0.76]
Patel 2018 [40]	1	348	3	112	3.2%	0.10 [0.01, 1.02]
Tahmasebi 2021 [41]	7	1710	6	314	7.1%	0.21 [0.07, 0.63]
Xu 2020 [42]	0	437	5	418	2.2%	0.09 [0.00, 1.56]
Yavuz 2020 [43]	4	474	5	502	6.1%	0.85 [0.23, 3.17]
**Total (95% CI)**		**31,023**		**40,119**	**100.0%**	**0.44 [0.27, 0.70]**
Total events	164		379			

Heterogeneity: Tau² = 0.51; Chi² = 51.47, df = 18 (P < 0.0001); I² = 65%
Test for overall effect: Z = 3.41 (P = 0.0006)

Figure S7. Forest Plot of Cohort Studies without Subgrouping by Administration Type

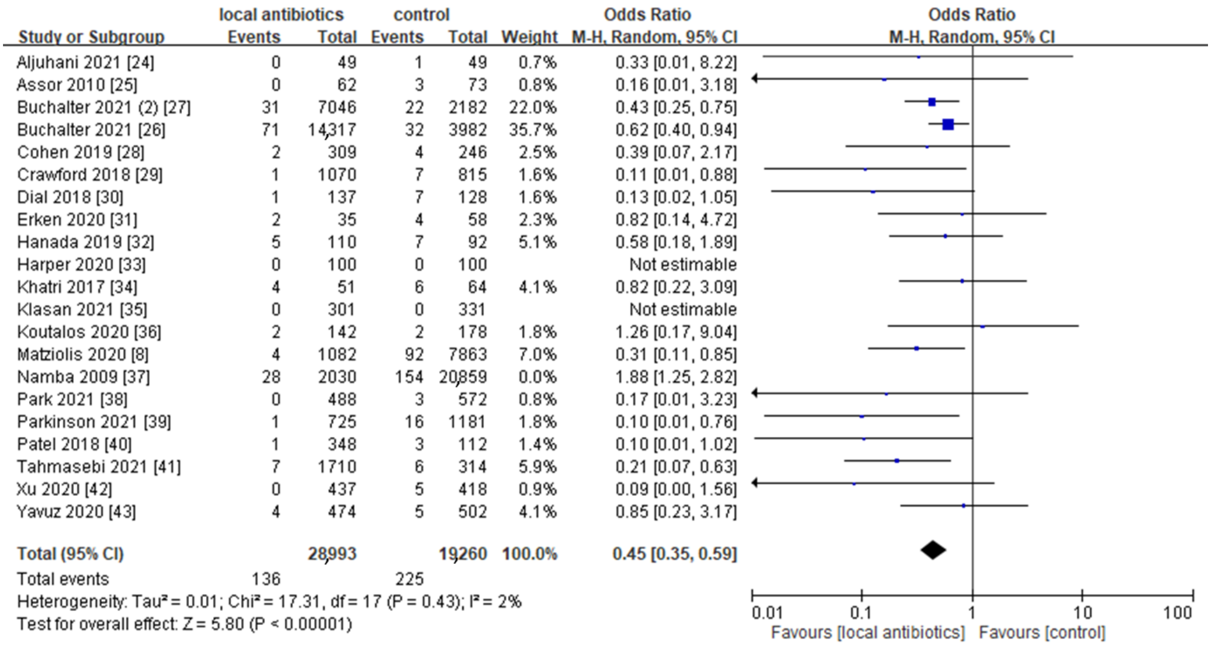

Figure S8. Sensitivity Analysis of Cohort Study without Subgroup Excluding Namba 2009 [37]

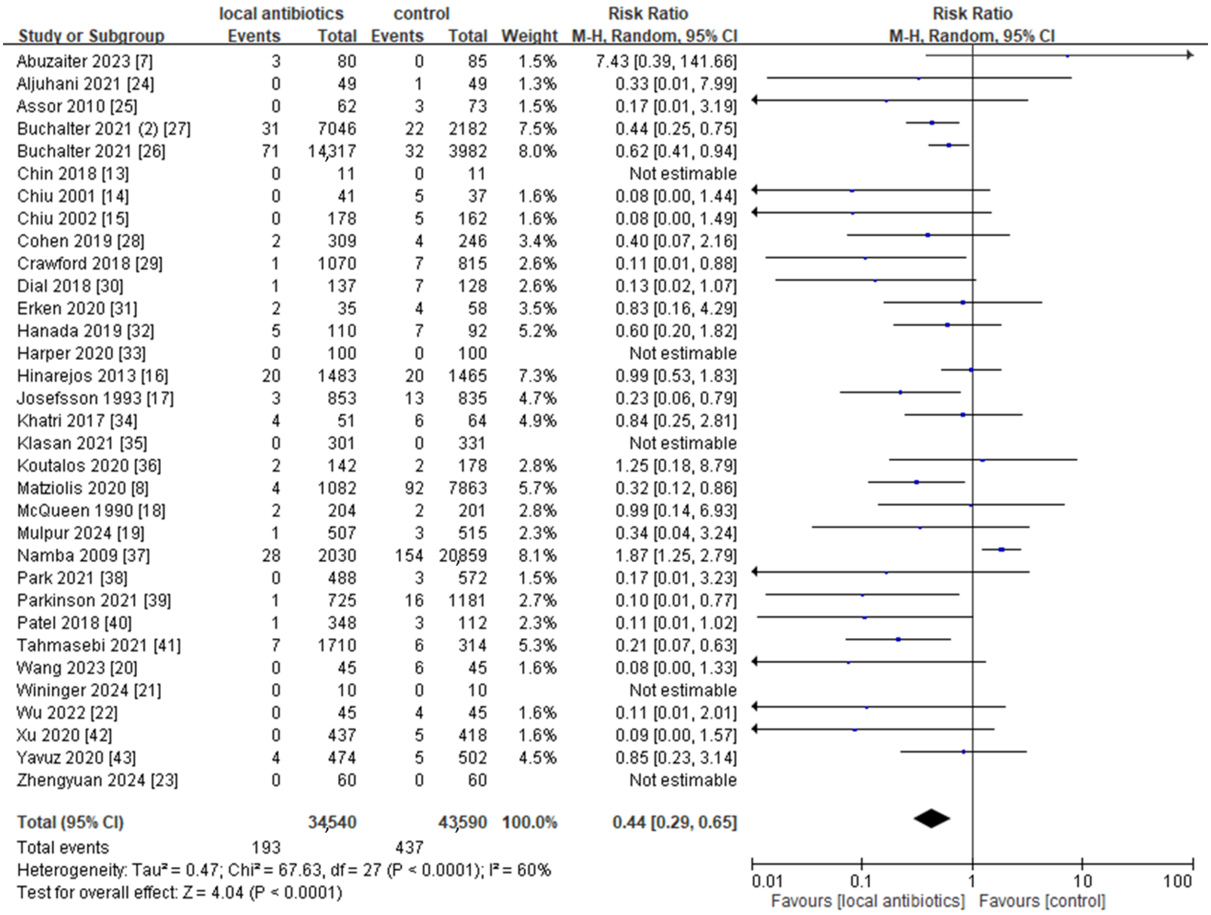

Figure S9. Forest Plot of Combined RCTs and Cohort Studies without Subgrouping by Administration Type

Study or Subgroup	local antibiotics Events	local antibiotics Total	control Events	control Total	Weight	Risk Ratio M-H, Random, 95% CI
Abuzaiter 2023 [7]	3	80	0	85	1.0%	7.43 [0.39, 141.66]
Aljuhani 2021 [24]	0	49	1	49	0.8%	0.33 [0.01, 7.99]
Assor 2010 [25]	0	62	3	73	1.0%	0.17 [0.01, 3.19]
Buchalter 2021 (2) [27]	31	7046	22	2182	16.6%	0.44 [0.25, 0.75]
Buchalter 2021 [26]	71	14,317	32	3982	21.7%	0.62 [0.41, 0.94]
Chin 2018 [13]	0	11	0	11		Not estimable
Chiu 2001 [14]	0	41	5	37	1.0%	0.08 [0.00, 1.44]
Chiu 2002 [15]	0	178	5	162	1.0%	0.08 [0.00, 1.49]
Cohen 2019 [28]	2	309	4	246	2.8%	0.40 [0.07, 2.16]
Crawford 2018 [29]	1	1070	7	815	1.9%	0.11 [0.01, 0.88]
Dial 2018 [30]	1	137	7	128	1.9%	0.13 [0.02, 1.07]
Erken 2020 [31]	2	35	4	58	3.0%	0.83 [0.16, 4.29]
Hanada 2019 [32]	5	110	7	92	5.9%	0.60 [0.20, 1.82]
Harper 2020 [33]	0	100	0	100		Not estimable
Hinarejos 2013 [16]	20	1483	20	1465	0.0%	0.99 [0.53, 1.83]
Josefsson 1993 [17]	3	853	13	835	4.8%	0.23 [0.06, 0.79]
Khatri 2017 [34]	4	51	6	64	5.1%	0.84 [0.25, 2.81]
Klasan 2021 [35]	0	301	0	331		Not estimable
Koutalos 2020 [36]	2	142	2	178	2.2%	1.25 [0.18, 8.79]
Matziolis 2020 [8]	4	1082	92	7863	7.1%	0.32 [0.12, 0.86]
McQueen 1990 [18]	2	204	2	201	2.2%	0.99 [0.14, 6.93]
Mulpur 2024 [19]	1	507	3	515	1.6%	0.34 [0.04, 3.24]
Namba 2009 [37]	28	2030	154	20,859	0.0%	1.87 [1.25, 2.79]
Park 2021 [38]	0	488	3	572	1.0%	0.17 [0.01, 3.23]
Parkinson 2021 [39]	1	725	16	1181	2.0%	0.10 [0.01, 0.77]
Patel 2018 [40]	1	348	3	112	1.6%	0.11 [0.01, 1.02]
Tahmasebi 2021 [41]	7	1710	6	314	6.2%	0.21 [0.07, 0.63]
Wang 2023 [20]	0	45	6	45	1.0%	0.08 [0.00, 1.33]
Wininger 2024 [21]	0	10	0	10		Not estimable
Wu 2022 [22]	0	45	4	45	1.0%	0.11 [0.01, 2.01]
Xu 2020 [42]	0	437	5	418	1.0%	0.09 [0.00, 1.57]
Yavuz 2020 [43]	4	474	5	502	4.5%	0.85 [0.23, 3.14]
Zhengyuan 2024 [23]	0	60	0	60		Not estimable
**Total (95% CI)**		**31,027**		**21,266**	**100.0%**	**0.41 [0.30, 0.55]**
Total events	145		263			

Heterogeneity: Tau² = 0.06; Chi² = 28.24, df = 25 (P = 0.30); I² = 11%

Test for overall effect: Z = 5.95 (P < 0.00001)

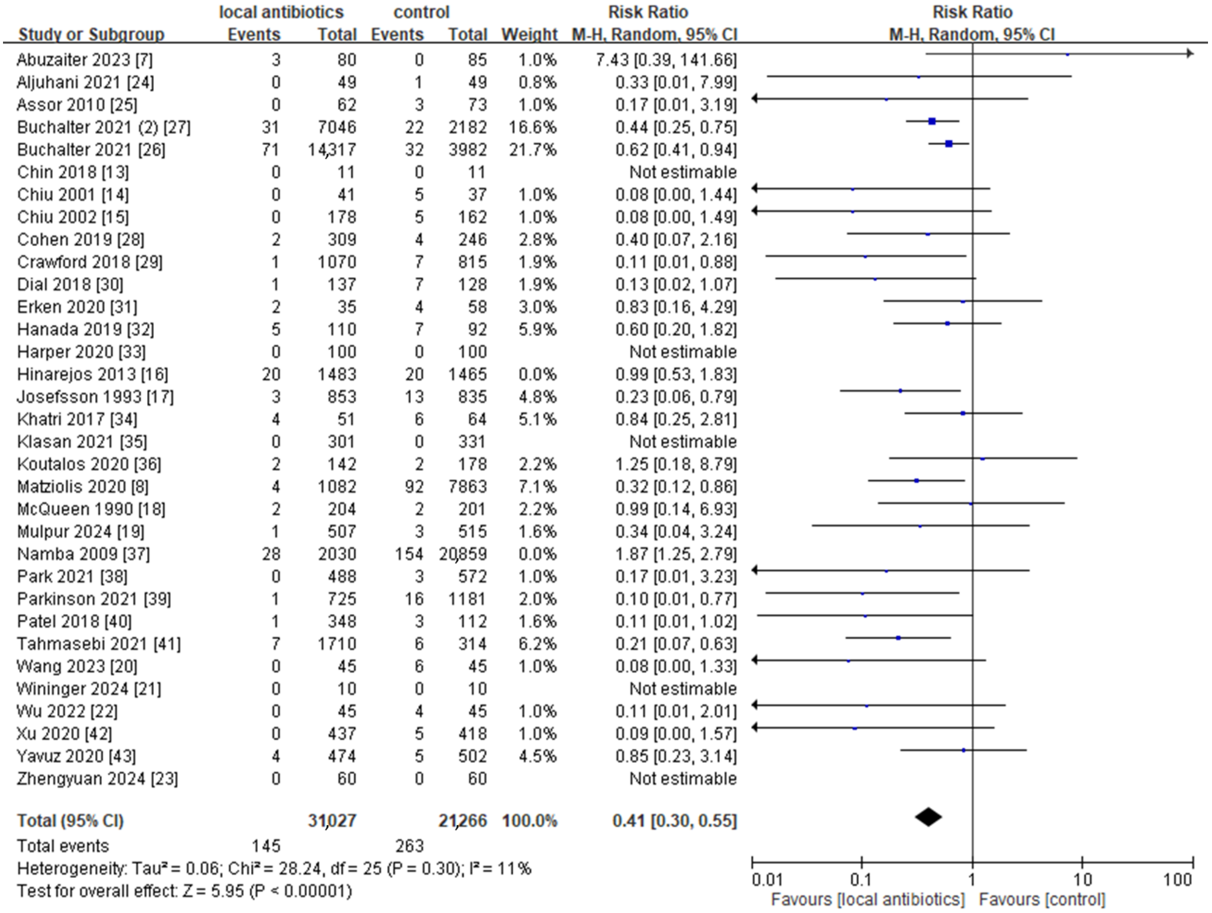

Figure S10. Sensitivity Analysis of RCT and Cohort Study without Subgroup Excluding Namba 2009 [37] and Hinarejos 2013 [16]

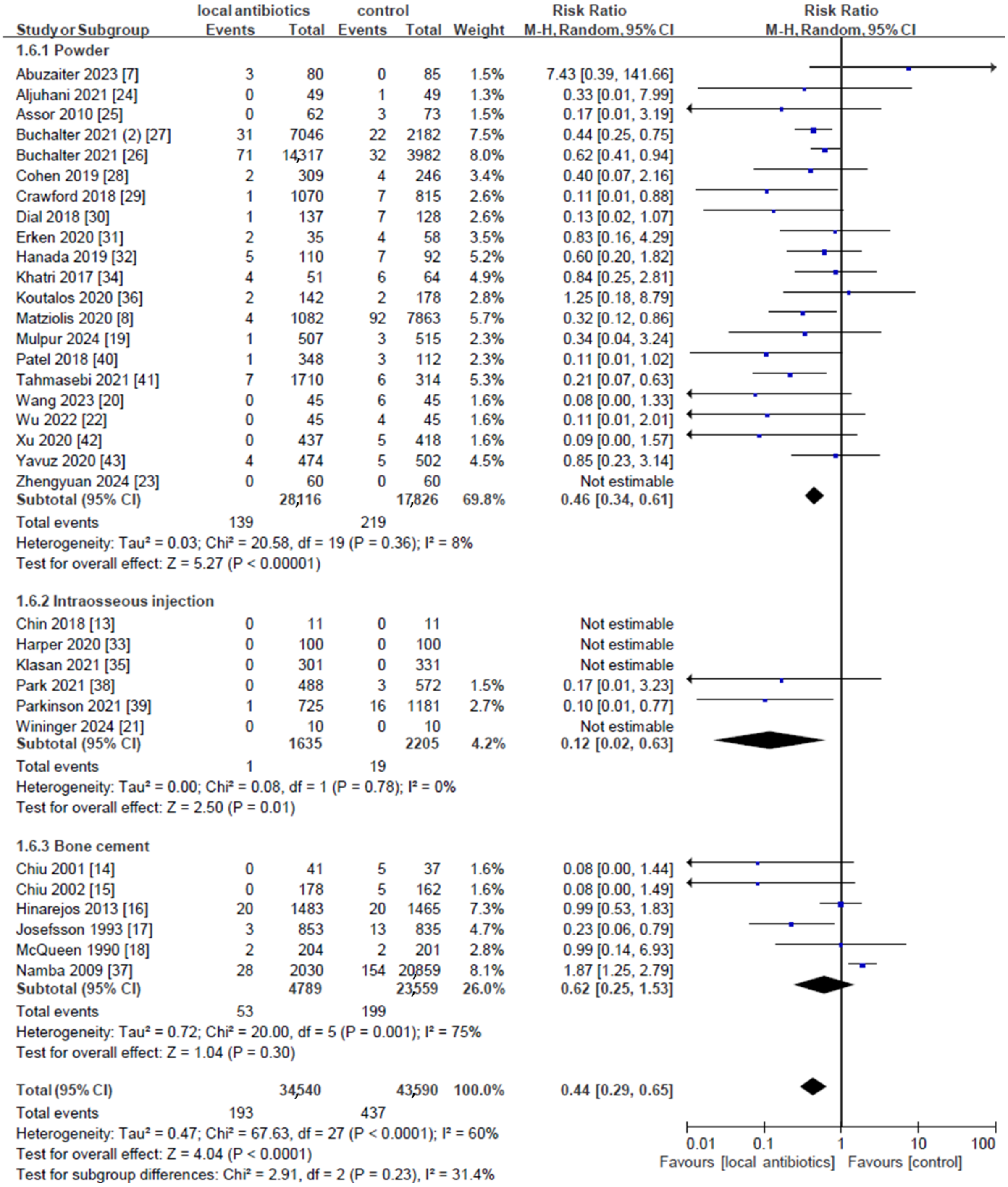

Figure S11. Forest Plot of Combined RCTs and Cohort Studies with Subgroup

Study or Subgroup	local antibiotics Events	local antibiotics Total	control Events	control Total	Weight	Risk Ratio M-H, Random, 95% CI
**1.6.1 Powder**						
Abuzaiter 2023 [7]	3	80	0	85	1.0%	7.43 [0.39, 141.66]
Aljuhani 2021 [24]	0	49	1	49	0.8%	0.33 [0.01, 7.99]
Assor 2010 [25]	0	62	3	73	1.0%	0.17 [0.01, 3.19]
Buchalter 2021 (2) [27]	31	7046	22	2182	16.6%	0.44 [0.25, 0.75]
Buchalter 2021 [26]	71	14,317	32	3982	21.7%	0.62 [0.41, 0.94]
Cohen 2019 [28]	2	309	4	246	2.8%	0.40 [0.07, 2.16]
Crawford 2018 [29]	1	1070	7	815	1.9%	0.11 [0.01, 0.88]
Dial 2018 [30]	1	137	7	128	1.9%	0.13 [0.02, 1.07]
Erken 2020 [31]	2	35	4	58	3.0%	0.83 [0.16, 4.29]
Hanada 2019 [32]	5	110	7	92	5.9%	0.60 [0.20, 1.82]
Khatri 2017 [34]	4	51	6	64	5.1%	0.84 [0.25, 2.81]
Koutalos 2020 [36]	2	142	2	178	2.2%	1.25 [0.18, 8.79]
Matziolis 2020 [8]	4	1082	92	7863	7.1%	0.32 [0.12, 0.86]
Mulpur 2024 [19]	1	507	3	515	1.6%	0.34 [0.04, 3.24]
Patel 2018 [40]	1	348	3	112	1.6%	0.11 [0.01, 1.02]
Tahmasebi 2021 [41]	7	1710	6	314	6.2%	0.21 [0.07, 0.63]
Wang 2023 [20]	0	45	6	45	1.0%	0.08 [0.00, 1.33]
Wu 2022 [22]	0	45	4	45	1.0%	0.11 [0.01, 2.01]
Xu 2020 [42]	0	437	5	418	1.0%	0.09 [0.00, 1.57]
Yavuz 2020 [43]	4	474	5	502	4.5%	0.85 [0.23, 3.14]
Zhengyuan 2024 [23]	0	60	0	60		Not estimable
**Subtotal (95% CI)**		**28,116**		**17,826**	**88.0%**	**0.46 [0.34, 0.61]**
Total events	139		219			
Heterogeneity: Tau² = 0.03; Chi² = 20.58, df = 19 (P = 0.36); I² = 8%						
Test for overall effect: Z = 5.27 (P < 0.00001)						
**1.6.2 Intraosseous injection**						
Chin 2018 [13]	0	11	0	11		Not estimable
Harper 2020 [33]	0	100	0	100		Not estimable
Klasan 2021 [35]	0	301	0	331		Not estimable
Park 2021 [38]	0	488	3	572	1.0%	0.17 [0.01, 3.23]
Parkinson 2021 [39]	1	725	16	1181	2.0%	0.10 [0.01, 0.77]
Wininger 2024 [21]	0	10	0	10		Not estimable
**Subtotal (95% CI)**		**1635**		**2205**	**3.0%**	**0.12 [0.02, 0.63]**
Total events	1		19			
Heterogeneity: Tau² = 0.00; Chi² = 0.08, df = 1 (P = 0.78); I² = 0%						
Test for overall effect: Z = 2.50 (P = 0.01)						
**1.6.3 Bone cement**						
Chiu 2001 [14]	0	41	5	37	1.0%	0.08 [0.00, 1.44]
Chiu 2002 [15]	0	178	5	162	1.0%	0.08 [0.00, 1.49]
Hinarejos 2013 [16]	20	1483	20	1465	0.0%	0.99 [0.53, 1.83]
Josefsson 1993 [17]	3	853	13	835	4.8%	0.23 [0.06, 0.79]
McQueen 1990 [18]	2	204	2	201	2.2%	0.99 [0.14, 6.93]
Namba 2009 [37]	28	2030	154	20,859	0.0%	1.87 [1.25, 2.79]
**Subtotal (95% CI)**		**1276**		**1235**	**9.0%**	**0.26 [0.10, 0.68]**
Total events	5		25			
Heterogeneity: Tau² = 0.06; Chi² = 3.16, df = 3 (P = 0.37); I² = 5%						
Test for overall effect: Z = 2.73 (P = 0.006)						
**Total (95% CI)**		**31,027**		**21,266**	**100.0%**	**0.41 [0.30, 0.55]**
Total events	145		263			
Heterogeneity: Tau² = 0.06; Chi² = 28.24, df = 25 (P = 0.30); I² = 11%						
Test for overall effect: Z = 5.95 (P < 0.00001)						
Test for subgroup differences: Chi² = 3.49, df = 2 (P = 0.17), I² = 42.6%						

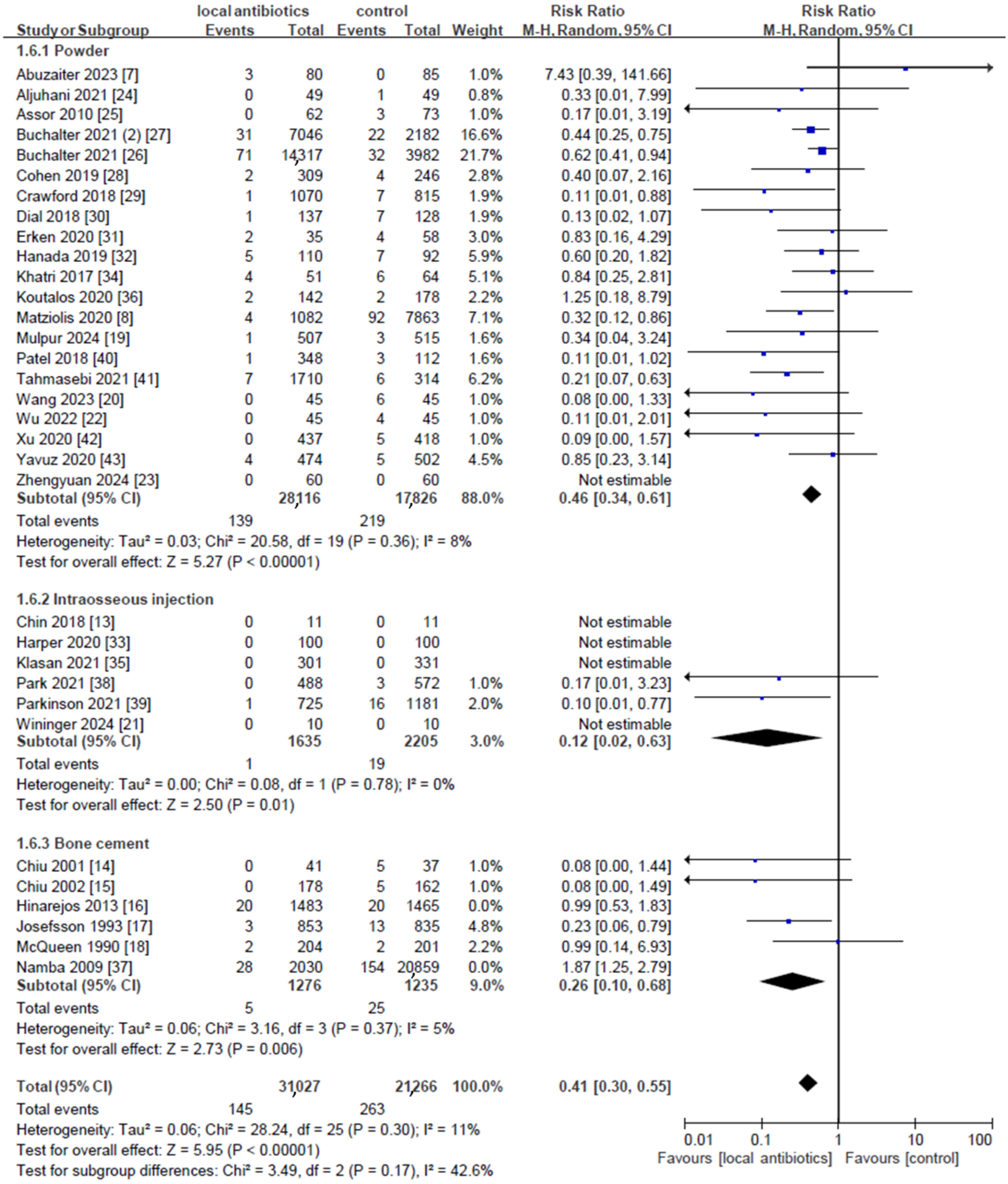

Figure S12. Sensitivity Analysis of RCT and Cohort Study with Subgroup Excluding Namba 2009 [37] and Hinarejos 2013 [16]

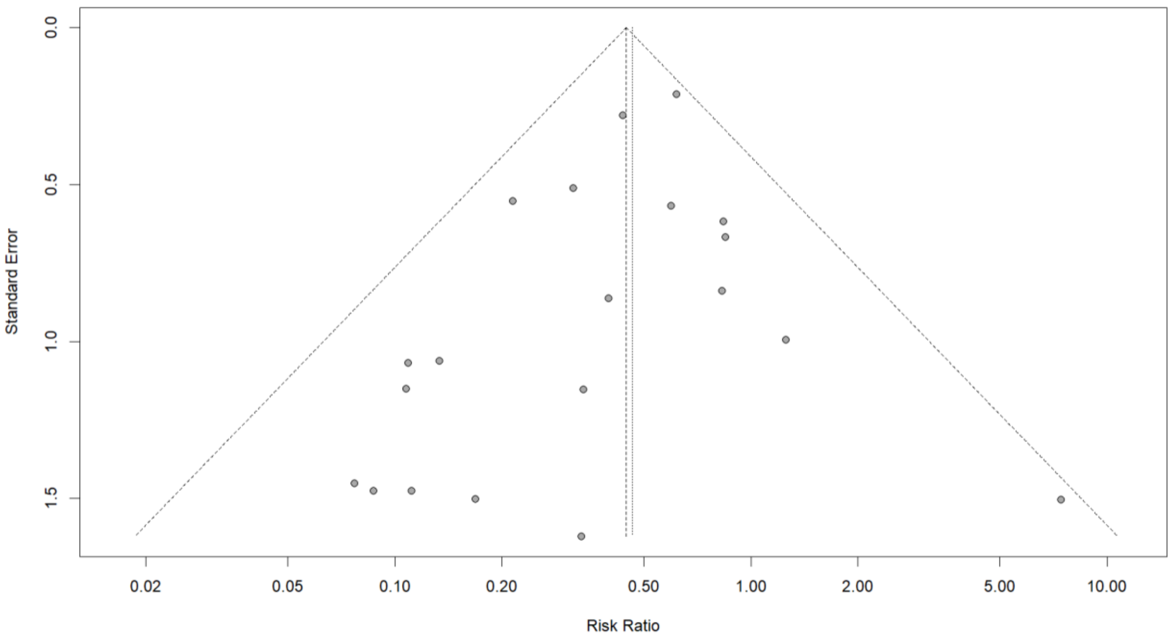

Figure S13. Funnel Plot of Powder Administration Data (Peters' test: p = 0.7487; Egger's test: p = 0.113)

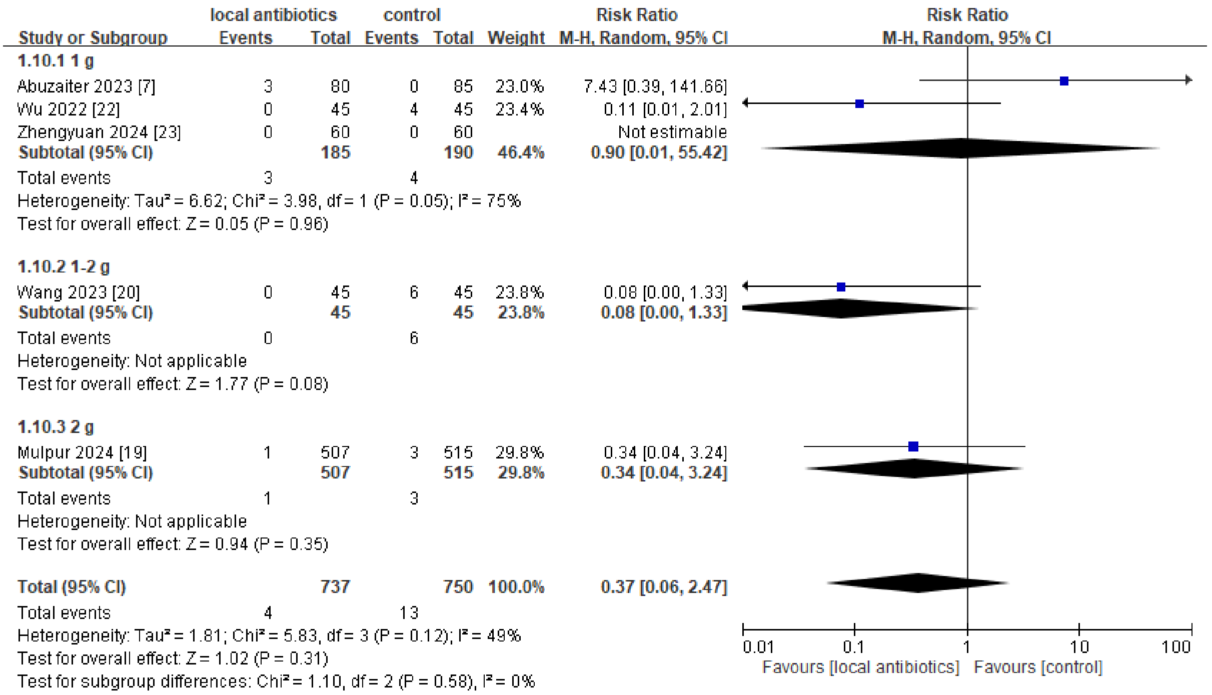

Figure S14. Forest Plot of RCTs Using Vancomycin Powder with Dosage Subgroup

Study or Subgroup	local antibiotics Events	local antibiotics Total	control Events	control Total	Weight	Odds Ratio M-H, Random, 95% CI
**1.11.1 1 g**						
Cohen 2019 [28]	2	309	4	246	2.4%	0.39 [0.07, 2.17]
Crawford 2018 [29]	1	1070	7	815	1.6%	0.11 [0.01, 0.88]
Dial 2018 [30]	1	137	7	128	1.5%	0.13 [0.02, 1.05]
Erken 2020 [31]	2	35	4	58	2.2%	0.82 [0.14, 4.72]
Hanada 2019 [32]	5	110	7	92	4.9%	0.58 [0.18, 1.89]
Khatri 2017 [34]	4	51	6	64	3.9%	0.82 [0.22, 3.09]
Matziolis 2020 [8]	4	1082	92	7863	6.8%	0.31 [0.11, 0.85]
Patel 2018 [40]	1	348	3	112	1.3%	0.10 [0.01, 1.02]
Tahmasebi 2021 [41]	7	1710	6	314	5.7%	0.21 [0.07, 0.63]
**Subtotal (95% CI)**		**4852**		**9692**	**30.3%**	**0.34 [0.21, 0.55]**
Total events	27		136			
Heterogeneity: Tau² = 0.00; Chi² = 7.27, df = 8 (P = 0.51); I² = 0%						
Test for overall effect: Z = 4.42 (P < 0.0001)						
**1.11.2 1-2 g**						
Assor 2010 [25]	0	62	3	73	0.8%	0.16 [0.01, 3.18]
**Subtotal (95% CI)**		**62**		**73**	**0.8%**	**0.16 [0.01, 3.18]**
Total events	0		3			
Heterogeneity: Not applicable						
Test for overall effect: Z = 1.20 (P = 0.23)						
**1.11.3 2 g**						
Aljuhani 2021 [24]	0	49	1	49	0.7%	0.33 [0.01, 8.22]
Buchalter 2021 (2) [27]	31	7046	22	2182	22.8%	0.43 [0.25, 0.75]
Buchalter 2021 [26]	71	14,317	32	3982	39.0%	0.62 [0.40, 0.94]
Koutalos 2020 [36]	2	142	2	178	1.8%	1.26 [0.17, 9.04]
Yavuz 2020 [43]	4	474	5	502	3.9%	0.85 [0.23, 3.17]
**Subtotal (95% CI)**		**22,028**		**6893**	**68.1%**	**0.56 [0.41, 0.78]**
Total events	108		62			
Heterogeneity: Tau² = 0.00; Chi² = 2.15, df = 4 (P = 0.71); I² = 0%						
Test for overall effect: Z = 3.54 (P = 0.0004)						
**1.11.4 0.5 g**						
Xu 2020 [42]	0	437	5	418	0.8%	0.09 [0.00, 1.56]
**Subtotal (95% CI)**		**437**		**418**	**0.8%**	**0.09 [0.00, 1.56]**
Total events	0		5			
Heterogeneity: Not applicable						
Test for overall effect: Z = 1.66 (P = 0.10)						
**Total (95% CI)**		**27,379**		**17,076**	**100.0%**	**0.47 [0.36, 0.61]**
Total events	135		206			
Heterogeneity: Tau² = 0.00; Chi² = 14.39, df = 15 (P = 0.50); I² = 0%						
Test for overall effect: Z = 5.60 (P < 0.00001)						
Test for subgroup differences: Chi² = 4.79, df = 3 (P = 0.19), I² = 37.4%						

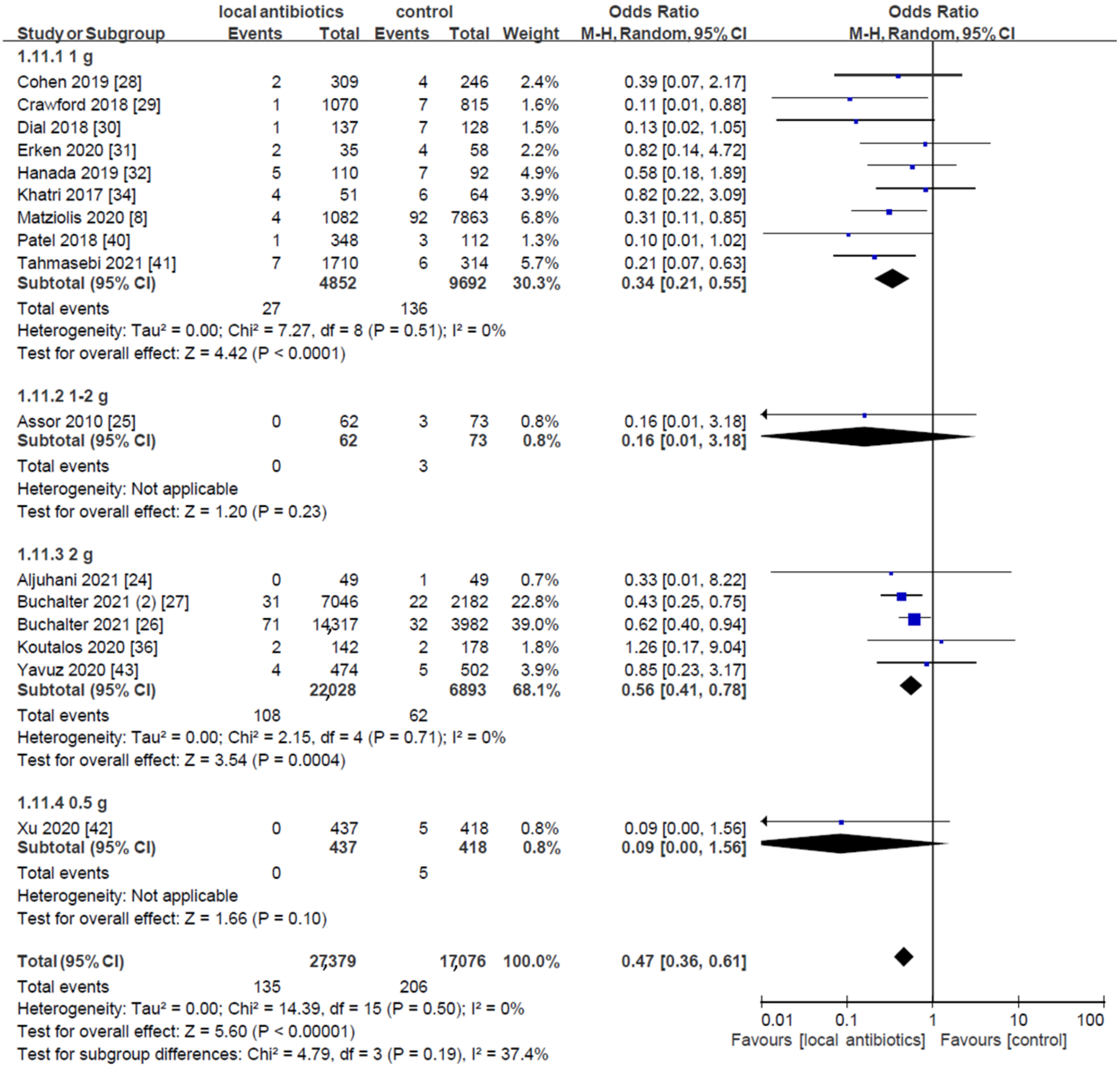

Figure S15. Forest Plot of Cohort Studies Using Vancomycin Powder with Dosage Subgroup

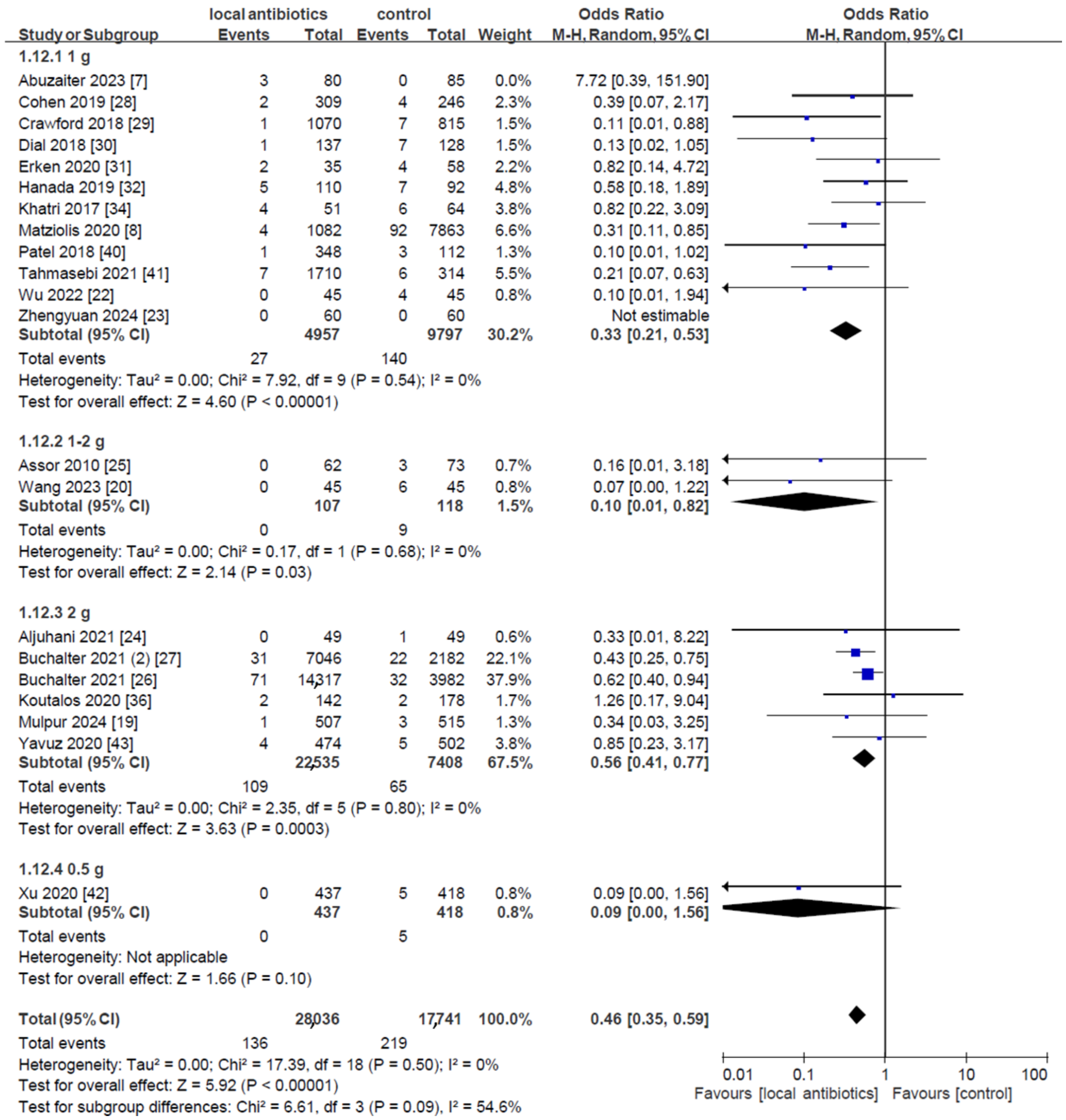

Figure S16. Sensitivity Analysis of RCT and Cohort Study with Dosage Subgroup Excluding Abuzaiter 2023 [7]
