# Supplementary material for: Effectiveness of Local Antibiotics for Infection Prevention in Primary Joint Arthroplasty: A Systematic Review and Meta-Analysis
Source: Antibiotics (Basel). 2025 Feb 20;14(3):214. doi: 10.3390/antibiotics14030214 (PMC11939600; doi:10.3390/antibiotics14030214)
Supplement: Supplementary file 1 [file antibiotics-14-00214-s001.zip › Supplementary Material File S2.pdf]

Supplementary Material File S2 (Meta-regression of Overall Data)

A. Administration type

1. Extracted data

Number	Study_ID	event.e	n.e	event.c	n.c	Administration
1	Abuzaiter 2023	3	80	0	85	powder
2	Aljuhani 2021	0	49	1	49	powder
3	Assor 2010	0	62	3	73	powder
4	Buchalter 2021	71	14317	32	3982	powder
5	Buchalter 2021 (2)	31	7046	22	2182	powder
6	Chin 2018	0	11	0	11	intraosseous
7	Chiu 2001	0	41	5	37	cement
8	Chiu 2002	0	178	5	162	cement
9	Cohen 2019	2	309	4	246	powder
10	Crawford 2018	1	1070	7	815	powder
11	Dial 2018	1	137	7	128	powder
12	Erken 2020	2	35	4	58	powder
13	Hanada 2019	5	110	7	92	powder
14	Harper 2020	0	100	0	100	intraosseous
15	Hinarejos 2013	20	1483	20	1465	cement
16	Josefsson 1993	3	853	13	835	cement
17	Khatri 2017	4	51	6	64	powder
18	Klasan 2021	0	301	0	331	intraosseous
19	Koutalos 2020	2	142	2	178	powder
20	Matziolis 2020	4	1082	92	7863	powder
21	McQueen 1990	2	204	2	201	cement
22	Mulpur 2024	1	507	3	515	powder
23	Namba 2009	28	2030	154	20859	cement
24	Park 2021	0	488	3	572	intraosseous
25	Parkinson 2021	1	725	16	1181	intraosseous
26	Patel 2018	1	348	3	112	powder
27	Tahmasebi 2021	7	1710	6	314	powder
28	Wang 2023	0	45	6	45	powder
29	Wininger 2024	0	10	0	10	intraosseous
30	Wu 2022	0	45	4	45	powder
31	Xu 2020	0	437	5	418	powder
32	Yavuz 2020	4	474	5	502	powder
33	Zhengyuan 2024	0	60	0	60	powder

2. Meta-regression

Moderators	Estimate	SE	Z value	P value	95%CI	
intrcpt	-0.2007	0.3092	-0.6489	0.5164	-0.8067	0.4054
intraosseous	-0.9529	0.7476	-1.2746	0.2024	-2.4180	0.5123
powder	-0.6549	0.3685	-1.7775	0.0755	-1.3771	0.0672

Mixed-effects model (k = 33; τ^2 = 0.2118 [estimated amount of residual heterogeneity]; I^2 = 33.35% [residual heterogeneity/unaccounted variability]; R^2 = 40.51% [amount of heterogeneity accounted for]; p = 0.0742, test for residual heterogeneity; p = 0.1611, test for moderators.

B. Study design

1. Extracted data

Number	Study_ID	event.e	n.e	event.c	n.c	Design
1	Abuzaiter 2023	3	80	0	85	RCT
2	Aljuhani 2021	0	49	1	49	Cohort
3	Assor 2010	0	62	3	73	Cohort
4	Buchalter 2021	71	14317	32	3982	Cohort
5	Buchalter 2021 (2)	31	7046	22	2182	Cohort
6	Chin 2018	0	11	0	11	RCT
7	Chiu 2001	0	41	5	37	RCT
8	Chiu 2002	0	178	5	162	RCT
9	Cohen 2019	2	309	4	246	Cohort
10	Crawford 2018	1	1070	7	815	Cohort
11	Dial 2018	1	137	7	128	Cohort
12	Erken 2020	2	35	4	58	Cohort
13	Hanada 2019	5	110	7	92	Cohort
14	Harper 2020	0	100	0	100	Cohort
15	Hinarejos 2013	20	1483	20	1465	RCT
16	Josefsson 1993	3	853	13	835	RCT
17	Khatri 2017	4	51	6	64	Cohort
18	Klasan 2021	0	301	0	331	Cohort
19	Koutalos 2020	2	142	2	178	Cohort
20	Matziolis 2020	4	1082	92	7863	Cohort
21	McQueen 1990	2	204	2	201	RCT
22	Mulpur 2024	1	507	3	515	RCT
23	Namba 2009	28	2030	154	20859	Cohort
24	Park 2021	0	488	3	572	Cohort
25	Parkinson 2021	1	725	16	1181	Cohort
26	Patel 2018	1	348	3	112	Cohort
27	Tahmasebi 2021	7	1710	6	314	Cohort
28	Wang 2023	0	45	6	45	RCT
29	Wininger 2024	0	10	0	10	RCT
30	Wu 2022	0	45	4	45	RCT
31	Xu 2020	0	437	5	418	Cohort
32	Yavuz 2020	4	474	5	502	Cohort
33	Zhengyuan 2024	0	60	0	60	RCT

2. Meta-regression

Moderators	Estimate	SE	Z value	P value	95%CI	
intrcpt	-0.7564	0.2185	-3.4613	0.0005*	-1.1847	-0.3281
DesignRCT	-0.0148	0.4291	-0.0345	0.9725	-0.8557	0.8262

Mixed-effects model (k = 33; τ^2 = 0.3807 [estimated amount of residual heterogeneity]; I^2 = 49.34% [residual heterogeneity/unaccounted variability]; R^2 = 0% [amount of heterogeneity accounted for]; p = 0.0003, test for residual heterogeneity; p = 0.9725, test for moderators.

*P<0.05, with statistical significance.

C. Age

1. Extracted data

Number	Study_ID	event.e	n.e	event.c	n.c	Age_ treatment	Age_ control	Age_ difference
1	Abuzaiter 2023	3	80	0	85	66	64	2
2	Assor 2010	0	62	3	73	73	72	1
3	Buchalter 2021	71	14317	32	3982	62.97	63.34	-0.37
4	Buchalter 2021 (2)	31	7046	22	2182	63.74	63.82	-0.08
5	Chin 2018	0	11	0	11	66	63	3
6	Chiu 2001	0	41	5	37	72	69	3
7	Chiu 2002	0	178	5	162	70	68	2
8	Cohen 2019	2	309	4	246	66	67.3	-1.3
9	Crawford 2018	1	1070	7	815	64.8	63.3	1.5
10	Dial 2018	1	137	7	128	61.2	61.5	-0.3
11	Erken 2020	2	35	4	58	81.88	81.87	0.01
12	Hanada 2019	5	110	7	92	74.6	73.3	1.3
13	Harper 2020	0	100	0	100	67	67	0
14	Hinarejos 2013	20	1483	20	1465	75.8	76.1	-0.3
15	Klasan 2021	0	301	0	331	67.7	68.7	-1
16	Matziolis 2020	4	1082	92	7863	69	68	1
17	McQueen 1990	2	204	2	201	67	67	0
18	Mulpur 2024	1	507	3	515	61.7	61.4	0.3
19	Park 2021	0	488	3	572	67.4	66.7	0.7
20	Parkinson 2021	1	725	16	1181	67	67	0
21	Patel 2018	1	348	3	112	63.6	64.9	-1.3
22	Tahmasebi 2021	7	1710	6	314	65	66.4	-1.4
23	Wang 2023	0	45	6	45	67.9	68	-0.1
24	Wininger 2024	0	10	0	10	69	67	2
25	Wu 2022	0	45	4	45	67.9	68	-0.1
26	Xu 2020	0	437	5	418	66.9	67.1	-0.2
27	Yavuz 2020	4	474	5	502	65.5	63.4	2.1
28	Zhengyuan 2024	0	60	0	60	68.3	66.5	1.8

2. Meta-regression

(1) Age_control+Age_difference

Moderators	Estimate	SE	Z value	P value	95%CI	
intrcpt	-3.7402	2.2669	-1.6499	0.099	-8.1833	0.7029
Age_control	0.0427	0.0334	1.2762	0.2019	-0.0229	0.1082
Age_difference	0.0731	0.1686	0.4336	0.6646	-0.2574	0.4036

Mixed-effects model (k = 28; τ^2 = 0.1455 [estimated amount of residual heterogeneity]; I^2 = 21.25% [residual heterogeneity/unaccounted variability]; R^2 = 0% [amount of heterogeneity accounted for]; p = 0.2965, test for residual heterogeneity; p = 0.4041, test for moderators.

(2) Age_difference

Moderators	Estimate	SE	Z value	P value	95%CI	
intrcpt	-0.8626	0.1769	-4.877	<0.0001*	-1.2093	-0.516
Age_difference	0.0743	0.1701	0.4371	0.6621	-0.259	0.4076

Mixed-effects model (k = 28; τ^2 = 0.1565 [estimated amount of residual heterogeneity]; I^2 = 25.15% [residual heterogeneity/unaccounted variability]; R^2 = 0% [amount of heterogeneity accounted for]; p = 0.2426, test for residual heterogeneity; p = 0.6621, test for moderators.

*P<0.05, with statistical significance.

(3) Age_treatment

Moderators	Estimate	SE	Z value	P value	95%CI	
intrcpt	-3.754	2.1482	-1.7475	0.0806	-7.9644	0.4564
Age_treatment	0.0432	0.0316	1.3661	0.1719	-0.0188	0.1052

Mixed-effects model (k = 28; τ^2 = 0.1211 [estimated amount of residual heterogeneity]; I^2 = 18.71% [residual heterogeneity/unaccounted variability]; R^2 = 3.67% [amount of heterogeneity accounted for]; p = 0.3432, test for residual heterogeneity; p = 0.1719, test for moderators.

D. Gender with male proportion

1. Extracted data

Number	Study_ID	event.e	n.e	event.c	n.c	Gender_ treatment	Gender_ control	Gender_ difference
1	Abuzaiter 2023	3	80	0	85	0.41	0.34	0.07
2	Aljuhani 2021	0	49	1	49	0.27	0.06	0.20
3	Assor 2010	0	62	3	73	0.26	0.23	0.03
4	Buchalter 2021	71	14317	32	3982	0.52	0.47	0.05
5	Buchalter 2021 (2)	31	7046	22	2182	0.39	0.55	-0.16
6	Chin 2018	0	11	0	11	0.64	0.55	0.09
7	Chiu 2001	0	41	5	37	0.68	0.67	0.01
8	Chiu 2002	0	178	5	162	0.70	0.60	0.10
9	Cohen 2019	2	309	4	246	0.48	0.44	0.04
10	Crawford 2018	1	1070	7	815	0.49	0.48	0.01
11	Dial 2018	1	137	7	128	0.47	0.50	-0.03
12	Erken 2020	2	35	4	58	0.48	0.52	-0.03
13	Hanada 2019	5	110	7	92	0.25	0.24	0.01
14	Harper 2020	0	100	0	100	0.47	0.40	0.07
15	Hinarejos 2013	20	1483	20	1465	0.23	0.24	-0.01
16	Khatri 2017	4	51	6	64	0.63	0.69	-0.06
17	Klasan 2021	0	301	0	331	0.42	0.43	-0.01
18	Koutalos 2020	2	142	2	178	0.29	0.28	0.01
19	Matziolis 2020	4	1082	92	7863	0.63	0.67	-0.04
20	McQueen 1990	2	204	2	201	0.42	0.33	0.08
21	Mulpur 2024	1	507	3	515	0.29	0.30	-0.01
22	Namba 2009	28	2030	154	20859	0.37	0.35	0.02
23	Park 2021	0	488	3	572	0.41	0.42	-0.01
24	Parkinson 2021	1	725	16	1181	0.48	0.51	-0.03
25	Patel 2018	1	348	3	112	0.40	0.43	-0.03
26	Tahmasebi 2021	7	1710	6	314	0.19	0.20	-0.01
27	Wang 2023	0	45	6	45	0.53	0.51	0.02
28	Wininger 2024	0	10	0	10	0.30	0.50	-0.20
29	Wu 2022	0	45	4	45	0.53	0.51	0.02
30	Xu 2020	0	437	5	418	0.28	0.31	-0.03
31	Yavuz 2020	4	474	5	502	0.31	0.31	0.01
32	Zhengyuan 2024	0	60	0	60	0.15	0.17	-0.02

2. Meta-regression

(1) Gender_control+Gender_difference

Moderators	Estimate	SE	Z value	P value	95%CI	
intrcpt	-0.0982	0.5943	-0.1652	0.8688	-1.2629	1.0665
Gender_control	-1.501	1.3748	-1.0918	0.2749	-4.1957	1.1936
Gender_difference	0.1616	3.2226	0.0502	0.96	-6.1546	6.4779

Mixed-effects model (k = 32; τ^2 = 0.3714 [estimated amount of residual heterogeneity]; I^2 = 44.79% [residual heterogeneity/unaccounted variability]; R^2 = 0% [amount of heterogeneity accounted for]; p = 0.004, test for residual heterogeneity; p = 0.4819, test for moderators.

(2) Gender_difference

Moderators	Estimate	SE	Z value	P value	95%CI	
intrcpt	-0.7086	0.1911	-3.7078	0.0002*	-1.0831	-0.334
Gender_difference	1.5506	2.9449	0.5265	0.5985	-4.2213	7.3225

Mixed-effects model (k = 32; τ^2 = 0.3615 [estimated amount of residual heterogeneity]; I^2 = 46.23% [residual heterogeneity/unaccounted variability]; R^2 = 0% [amount of heterogeneity accounted for]; p = 0.0016, test for residual heterogeneity; p = 0.5985, test for moderators.

*P<0.05, with statistical significance.

(3) Gender_treatment

Moderators	Estimate	SE	Z value	P value	95%CI	
intrcpt	-0.1209	0.5881	-0.2055	0.8372	-1.2736	1.0318
Gender_treatment	-1.466	1.3637	-1.075	0.2824	-4.1388	1.2068

Mixed-effects model (k = 32; τ^2 = 0.3599 [estimated amount of residual heterogeneity]; I^2 = 47.65% [residual heterogeneity/unaccounted variability]; R^2 = 0% [amount of heterogeneity accounted for]; p = 0.0012, test for residual heterogeneity; p = 0.2824, test for moderators.

E. Diagnosis of diabetes mellitus proportion

1. Extracted data

Number	Study_ID	event.e	n.e	event.c	n.c	DM_ treatment	DM_ control	DM_ difference
1	Abuzaiter 2023	3	80	0	85	0.20	0.15	0.05
2	Buchalter 2021	71	14317	32	3982	0.08	0.22	-0.13
3	Buchalter 2021 (2)	31	7046	22	2182	0.07	0.23	-0.16
4	Chiu 2001	0	41	5	37	1.00	1.00	0.00
5	Crawford 2018	1	1070	7	815	0.14	0.16	-0.02
6	Dial 2018	1	137	7	128	0.12	0.15	-0.03
7	Hanada 2019	5	110	7	92	0.20	0.19	0.02
8	Harper 2020	0	100	0	100	0.26	0.20	0.06
9	Hinarejos 2013	20	1483	20	1465	0.17	0.18	-0.01
10	Khatri 2017	4	51	6	64	0.33	0.36	-0.03
11	Klasan 2021	0	301	0	331	0.13	0.10	0.02
12	Mulpur 2024	1	507	3	515	0.34	0.31	0.02
13	Namba 2009	28	2030	154	20859	0.15	0.10	0.04
14	Park 2021	0	488	3	572	0.23	0.23	0.00
15	Parkinson 2021	1	725	16	1181	0.09	0.14	-0.05
16	Patel 2018	1	348	3	112	0.10	0.13	-0.03
17	Xu 2020	0	437	5	418	0.10	0.14	-0.04
18	Yavuz 2020	4	474	5	502	0.22	0.26	-0.04

2. Meta-regression

(1) DM_control+DM_difference

Moderators	Estimate	SE	Z value	P value	95%CI	
intrcpt	-0.0433	0.431	-0.1005	0.9199	-0.888	0.8014
DM_control	-1.7658	1.6971	-1.0405	0.2981	-5.0919	1.5604
DM_difference	3.6513	3.1973	1.142	0.2535	-2.6153	9.918

Mixed-effects model (k = 18; τ^2 = 0.3131 [estimated amount of residual heterogeneity]; I^2 = 48.98% [residual heterogeneity/unaccounted variability]; R^2 = 25.72% [amount of heterogeneity accounted for]; p = 0.0454, test for residual heterogeneity; p = 0.2598, test for moderators.

(2) DM_difference

Moderators	Estimate	SE	Z value	P value	95%CI	
intrcpt	-0.4388	0.27	-1.6252	0.1041	-0.9681	0.0904
DM_difference	3.9983	3.4099	1.1726	0.241	-2.6849	10.6815

Mixed-effects model (k = 18; τ^2 = 0.3777 [estimated amount of residual heterogeneity]; I^2 = 53.49% [residual heterogeneity/unaccounted variability]; R^2 = 10.39% [amount of heterogeneity accounted for]; p = 0.0234, test for residual heterogeneity; p = 0.241, test for moderators.

(3) DM_treatment

Moderators	Estimate	SE	Z value	P value	95%CI	
intrcpt	-0.4867	0.3851	-1.2638	0.2063	-1.2414	0.2681
DM_treatment	-0.7775	1.6499	-0.4712	0.6375	-4.0112	2.4562

Mixed-effects model (k = 18; τ^2 = 0.4577 [estimated amount of residual heterogeneity]; I^2 = 64.43% [residual heterogeneity/unaccounted variability]; R^2 = 0% [amount of heterogeneity accounted for]; p <0.0001, test for residual heterogeneity; p = 0.6375, test for moderators.

F. BMI

1. Extracted data

Number	Study_ID	event.e	n.e	event.c	n.c	BMI_ treatment	BMI_ control	BMI_ difference
1	Abuzaiter 2023	3	80	0	85	33.4	35.7	-2.3
2	Buchalter 2021	71	14317	32	3982	32.9	34.1	-1.2
3	Buchalter 2021 (2)	31	7046	22	2182	34.02	35.06	-1.04
4	Chin 2018	0	11	0	11	41	40	1
5	Crawford 2018	1	1070	7	815	31	31.1	-0.1
6	Dial 2018	1	137	7	128	30	29.8	0.2
7	Hanada 2019	5	110	7	92	26.7	25.7	1
8	Harper 2020	0	100	0	100	32	32	0
9	Hinarejos 2013	20	1483	20	1465	31.5	31.74	-0.24
10	Klasan 2021	0	301	0	331	31.8	31.4	0.4
11	Matziolis 2020	4	1082	92	7863	29.5	29.8	-0.3
12	Mulpur 2024	1	507	3	515	28.5	28.4	0.1
13	Park 2021	0	488	3	572	31.99	32.47	-0.48
14	Parkinson 2021	1	725	16	1181	31.5	31.3	0.2
15	Patel 2018	1	348	3	112	30.6	31.1	-0.5
16	Wininger 2024	0	10	0	10	29.38	30.97	-1.59
17	Xu 2020	0	437	5	418	25.3	24.9	0.4
18	Yavuz 2020	4	474	5	502	29	28.9	0.1
19	Zhengyuan 2024	0	60	0	60	25.8	26.2	-0.4

2. Meta-regression

(1) BMI_control+BMI_difference

Moderators	Estimate	SE	Z value	P value	95%CI	
intrcpt	-1.1784	3.2141	-0.3666	0.7139	-7.4779	5.1211
BMI_control	-0.0111	0.106	0.1043	0.9169	-0.1966	0.2187
BMI_difference	-0.2337	0.449	-0.5205	0.6027	-1.1137	0.6463

Mixed-effects model (k = 19; τ^2 = 0.1774 [estimated amount of residual heterogeneity]; I^2 = 28.88% [residual heterogeneity/unaccounted variability]; R^2 = 0% [amount of heterogeneity accounted for]; p = 0.2106, test for residual heterogeneity; p = 0.6301, test for moderators.

(2) BMI_difference

Moderators	Estimate	SE	Z value	P value	95%CI	
intrcpt	-0.8332	0.2302	-3.6189	0.0003*	-1.2845	-0.382
BMI_difference	-0.2588	0.2748	-0.9417	0.3464	-0.7975	0.2799

Mixed-effects model (k = 19; τ^2 = 0.1543 [estimated amount of residual heterogeneity]; I^2 = 26.67% [residual heterogeneity/unaccounted variability]; R^2 = 0% [amount of heterogeneity accounted for]; p = 0.2631, test for residual heterogeneity; p = 0.3464, test for moderators.
*P<0.05, with statistical significance.

(3) BMI_treatment

Moderators	Estimate	SE	Z value	P value	95%CI	
intrcpt	-2.3547	2.3762	-0.9909	0.3217	-7.0119	2.3026
BMI_treatment	0.0526	0.0756	-0.696	0.4864	-0.0956	0.2008

Mixed-effects model (k = 19; τ^2 = 0.1225 [estimated amount of residual heterogeneity]; I^2 = 23.49% [residual heterogeneity/unaccounted variability]; R^2 = 0% [amount of heterogeneity accounted for]; p = 0.2549, test for residual heterogeneity; p = 0.4864, test for moderators.

G. Diagnosis of rheumatic arthritis

1. Extracted data

Number	Study_ID	event.e	n.e	event.c	n.c	RA_ treatment	RA_ control	RA_ difference
1	Buchalter 2021	71	14317	32	3982	0.07	0.03	0.04
2	Buchalter 2021 (2)	31	7046	22	2182	0.13	0.00	0.13
3	Crawford 2018	1	1070	7	815	0.06	0.03	0.04
4	Dial 2018	1	137	7	128	0.07	0.06	0.01
5	Harper 2020	0	100	0	100	0.02	0.03	-0.01
6	Park 2021	0	488	3	572	0.06	0.06	0.00

2. Meta-regression

(1) RA_control+RA_difference

Moderators	Estimate	SE	Z value	P value	95%CI	
intrcpt	0.7366	2.9473	-0.2499	0.8026	-5.04	6.5132
RA_control	-41.2535	61.1409	-0.6747	0.4998	-161.0874	78.5805
RA_difference	-11.8583	23.9568	-0.495	0.6206	-58.8128	35.0962

Mixed-effects model (k = 6; $\tau^2 = 0.3858$ [estimated amount of residual heterogeneity]; $I^2 = 38.89\%$ [residual heterogeneity/unaccounted variability]; $R^2 = 0\%$ [amount of heterogeneity accounted for]; p = 0.2266, test for residual heterogeneity; p = 0.7286, test for moderators.

(2) RA_difference

Moderators	Estimate	SE	Z value	P value	95%CI	
intrcpt	-1.1923	0.6279	-1.8989	0.0576	-2.423	0.0384
RA_difference	-3.2873	7.8593	-0.4183	0.6757	-12.1166	18.6913

Mixed-effects model (k = 6; $\tau^2 = 0.364$ [estimated amount of residual heterogeneity]; $I^2 = 34.73\%$ [residual heterogeneity/unaccounted variability]; $R^2 = 0\%$ [amount of heterogeneity accounted for]; p = 0.2605, test for residual heterogeneity; p = 0.6757, test for moderators.

(3) RA_treatment

Moderators	Estimate	SE	Z value	P value	95%CI	
intrcpt	-1.2613	1.1129	-1.1334	0.2571	-3.4425	0.9199
RA_treatment	3.2509	12.0253	0.2703	0.7869	-20.3182	26.82

Mixed-effects model (k = 6; $\tau^2 = 0.3473$ [estimated amount of residual heterogeneity]; $I^2 = 33\%$ [residual heterogeneity/unaccounted variability]; $R^2 = 0\%$ [amount of heterogeneity accounted for]; p = 0.2695, test for residual heterogeneity; p = 0.7869, test for moderators.

H. Smoking

1. Extracted data

Number	Study_ID	event.e	n.e	event.c	n.c	Smoking_ treatment	Smoking_ control	Smoking_ difference
1	Abuzaiter 2023	3	80	0	85	0.11	0.05	0.07
2	Dial 2018	1	137	7	128	0.16	0.15	0.01
3	Khatri 2017	4	51	6	64	0.12	0.08	0.04
4	Mulpur 2024	1	507	3	515	0.11	0.12	0.00
5	Park 2021	0	488	3	572	0.05	0.05	0.00
6	Parkinson 2021	1	725	16	1181	0.04	0.02	0.02
7	Patel 2018	1	348	3	112	0.11	0.13	-0.02
8	Xu 2020	0	437	5	418	0.22	0.21	0.01

2. Meta-regression

(1) Smoking_control+Smoking_difference

Moderators	Estimate	SE	Z value	P value	95%CI	
intrcpt	-2.0481	1.0782	-1.8995	0.0575	-4.1614	0.0652
Smoking_control	-0.0345	8.3745	-0.0041	0.9967	-16.4482	16.3792
Smoking _difference	44.6953	18.6953	2.3907	0.0168*	-8.0531	81.3375

Mixed-effects model (k = 8; $\tau^2 = 0$ [estimated amount of residual heterogeneity]; $I^2 = 0\%$ [residual heterogeneity/unaccounted variability]; $R^2 = 100\%$ [amount of heterogeneity accounted for]; p = 0.6081, test for residual heterogeneity; p = 0.0308, test for moderators.

*P<0.05, with statistical significance.

(2) Smoking_difference

Moderators	Estimate	SE	Z value	P value	95%CI	
intrcpt	-2.052	0.5206	-3.9417	<0.0001*	-3.0724	-1.0317
Smoking_difference	44.7277	16.9566	2.6378	0.0083*	11.4934	77.962

Mixed-effects model (k = 8; $\tau^2 = 0$ [estimated amount of residual heterogeneity]; $I^2 = 0\%$ [residual heterogeneity/unaccounted variability]; $R^2 = 100\%$ [amount of heterogeneity accounted for]; p = 0.7304, test for residual heterogeneity; p = 0.0083, test for moderators.

*P<0.05, with statistical significance.

(3) Smoking_treatment

Moderators	Estimate	SE	Z value	P value	95%CI	
intrcpt	-1.1694	1.2815	-0.9126	0.3615	-3.681	1.3421
Smoking_treatment	-0.5563	10.2968	-0.054	0.9569	-20.7376	19.625

Mixed-effects model (k = 8; $\tau^2 = 0.7701$ [estimated amount of residual heterogeneity]; $I^2 = 40\%$

[residual heterogeneity/unaccounted variability]; R^2 = 0% [amount of heterogeneity accounted for]; p = 0.1032, test for residual heterogeneity; p = 0.9569, test for moderators.
