# Supplementary material for: Effectiveness of Local Antibiotics for Infection Prevention in Primary Joint Arthroplasty: A Systematic Review and Meta-Analysis
Source: Antibiotics (Basel). 2025 Feb 20;14(3):214. doi: 10.3390/antibiotics14030214 (PMC11939600; doi:10.3390/antibiotics14030214)
Supplement: Supplementary file 1 [file antibiotics-14-00214-s001.zip › Supplementary Material File S3.pdf]

Supplementary Material File S3 (Meta-regression of Overall Data without Hinarejos 2013 [16] and Namba 2009 [37])

A. Administration type

1. Extracted data

Number	Study_ID	event.e	n.e	event.c	n.c	Administration
1	Abuzaiter 2023	3	80	0	85	powder
2	Aljuhani 2021	0	49	1	49	powder
3	Assor 2010	0	62	3	73	powder
4	Buchalter 2021	71	14317	32	3982	powder
5	Buchalter 2021 (2)	31	7046	22	2182	powder
6	Chin 2018	0	11	0	11	intraosseous
7	Chiu 2001	0	41	5	37	cement
8	Chiu 2002	0	178	5	162	cement
9	Cohen 2019	2	309	4	246	powder
10	Crawford 2018	1	1070	7	815	powder
11	Dial 2018	1	137	7	128	powder
12	Erken 2020	2	35	4	58	powder
13	Hanada 2019	5	110	7	92	powder
14	Harper 2020	0	100	0	100	intraosseous
15	Josefsson 1993	3	853	13	835	cement
16	Khatri 2017	4	51	6	64	powder
17	Klasan 2021	0	301	0	331	intraosseous
18	Koutalos 2020	2	142	2	178	powder
19	Matziolis 2020	4	1082	92	7863	powder
20	McQueen 1990	2	204	2	201	cement
21	Mulpur 2024	1	507	3	515	powder
22	Park 2021	0	488	3	572	intraosseous
23	Parkinson 2021	1	725	16	1181	intraosseous
24	Patel 2018	1	348	3	112	powder
25	Tahmasebi 2021	7	1710	6	314	powder
26	Wang 2023	0	45	6	45	powder
27	Wininger 2024	0	10	0	10	intraosseous
28	Wu 2022	0	45	4	45	powder
29	Xu 2020	0	437	5	418	powder
30	Yavuz 2020	4	474	5	502	powder
31	Zhengyuan 2024	0	60	0	60	powder

2. Meta-regression

**Moderators**	**Estimate**	**SE**	**Z value**	**P value**	**95%CI**	
intrcpt	-1.3627	0.4831	-2.8207	0.0048*	-2.3095	-0.4158
intraosseous	0.1558	0.8078	0.1929	0.847	-1.4274	1.739
powder	-0.6012	0.5027	1.1961	0.2317	-0.384	1.5864

Mixed-effects model (k = 31; τ^2 = 0.0148 [estimated amount of residual heterogeneity]; I^2 = 2.71% [residual heterogeneity/unaccounted variability]; R^2 = 60.62% [amount of heterogeneity accounted for]; p = 0.5541, test for residual heterogeneity; p = 0.4074, test for moderators.

*P<0.05, with statistical significance.

B. Study design

1. Extracted data

Number	Study_ID	event.e	n.e	event.c	n.c	Design
1	Abuzaiter 2023	3	80	0	85	RCT
2	Aljuhani 2021	0	49	1	49	Cohort
3	Assor 2010	0	62	3	73	Cohort
4	Buchalter 2021	71	14317	32	3982	Cohort
5	Buchalter 2021 (2)	31	7046	22	2182	Cohort
6	Chin 2018	0	11	0	11	RCT
7	Chiu 2001	0	41	5	37	RCT
8	Chiu 2002	0	178	5	162	RCT
9	Cohen 2019	2	309	4	246	Cohort
10	Crawford 2018	1	1070	7	815	Cohort
11	Dial 2018	1	137	7	128	Cohort
12	Erken 2020	2	35	4	58	Cohort
13	Hanada 2019	5	110	7	92	Cohort
14	Harper 2020	0	100	0	100	Cohort
15	Josefsson 1993	3	853	13	835	RCT
16	Khatri 2017	4	51	6	64	Cohort
17	Klasan 2021	0	301	0	331	Cohort
18	Koutalos 2020	2	142	2	178	Cohort
19	Matziolis 2020	4	1082	92	7863	Cohort
20	McQueen 1990	2	204	2	201	RCT
21	Mulpur 2024	1	507	3	515	RCT
22	Park 2021	0	488	3	572	Cohort
23	Parkinson 2021	1	725	16	1181	Cohort
24	Patel 2018	1	348	3	112	Cohort
25	Tahmasebi 2021	7	1710	6	314	Cohort
26	Wang 2023	0	45	6	45	RCT
27	Wininger 2024	0	10	0	10	RCT
28	Wu 2022	0	45	4	45	RCT
29	Xu 2020	0	437	5	418	Cohort
30	Yavuz 2020	4	474	5	502	Cohort
31	Zhengyuan 2024	0	60	0	60	RCT

2. Meta-regression

**Moderators**	**Estimate**	**SE**	**Z value**	**P value**	**95%CI**	
intrcpt	-0.7951	0.1447	-5.4952	<0.0001*	-1.0786	-0.5115
DesignRCT	-0.3157	0.4024	-0.7847	0.4327	-1.1045	0.473

Mixed-effects model (k = 31; τ^2 = 0.0237 [estimated amount of residual heterogeneity]; I^2 = 4.22% [residual heterogeneity/unaccounted variability]; R^2 = 36.67% [amount of heterogeneity accounted for]; p = 0.543, test for residual heterogeneity; p = 0.4327, test for moderators.

*P<0.05, with statistical significance.

C. Age

1. Extracted data

Number	Study_ID	event.e	n.e	event.c	n.c	Age_ treatment	Age_ control	Age_ difference
1	Abuzaiter 2023	3	80	0	85	66	64	2
2	Assor 2010	0	62	3	73	73	72	1
3	Buchalter 2021	71	14317	32	3982	62.97	63.34	-0.37
4	Buchalter 2021 (2)	31	7046	22	2182	63.74	63.82	-0.08
5	Chin 2018	0	11	0	11	66	63	3
6	Chiu 2001	0	41	5	37	72	69	3
7	Chiu 2002	0	178	5	162	70	68	2
8	Cohen 2019	2	309	4	246	66	67.3	-1.3
9	Crawford 2018	1	1070	7	815	64.8	63.3	1.5
10	Dial 2018	1	137	7	128	61.2	61.5	-0.3
11	Erken 2020	2	35	4	58	81.88	81.87	0.01
12	Hanada 2019	5	110	7	92	74.6	73.3	1.3
13	Harper 2020	0	100	0	100	67	67	0
14	Klasan 2021	0	301	0	331	67.7	68.7	-1
15	Matziolis 2020	4	1082	92	7863	69	68	1
16	McQueen 1990	2	204	2	201	67	67	0
17	Mulpur 2024	1	507	3	515	61.7	61.4	0.3
18	Park 2021	0	488	3	572	67.4	66.7	0.7
19	Parkinson 2021	1	725	16	1181	67	67	0
20	Patel 2018	1	348	3	112	63.6	64.9	-1.3
21	Tahmasebi 2021	7	1710	6	314	65	66.4	-1.4
22	Wang 2023	0	45	6	45	67.9	68	-0.1
23	Wininger 2024	0	10	0	10	69	67	2
24	Wu 2022	0	45	4	45	67.9	68	-0.1
25	Xu 2020	0	437	5	418	66.9	67.1	-0.2
26	Yavuz 2020	4	474	5	502	65.5	63.4	2.1
27	Zhengyuan 2024	0	60	0	60	68.3	66.5	1.8

2. Meta-regression

(1) Age_control+Age_difference

Moderators	Estimate	SE	Z value	P value	95%CI	
intrcpt	-1.0706	2.7187	-0.3938	0.6937	-6.3993	4.258
Age_control	0.0017	0.041	0.0415	0.9669	-0.0787	0.082
Age_difference	0.1185	0.1669	0.7099	0.4778	-0.2087	0.4456

Mixed-effects model (k = 27; τ^2 = 0.1013 [estimated amount of residual heterogeneity]; I^2 = 14.91% [residual heterogeneity/unaccounted variability]; R^2 = 0% [amount of heterogeneity accounted for]; p = 0.4211, test for residual heterogeneity; p = 0.7684, test for moderators.

(2) Age_difference

Moderators	Estimate	SE	Z value	P value	95%CI	
intrcpt	-0.9403	0.1687	-5.5731	<0.0001*	-1.271	-0.6096
Age_difference	0.1137	0.1618	0.703	0.4821	-0.2033	0.4308

Mixed-effects model (k = 27; τ^2 = 0.0807 [estimated amount of residual heterogeneity]; I^2 = 12.72% [residual heterogeneity/unaccounted variability]; R^2 = 0% [amount of heterogeneity accounted for]; p = 0.4683, test for residual heterogeneity; p = 0.4821, test for moderators.

*P<0.05, with statistical significance.

(3) Age_treatment

Moderators	Estimate	SE	Z value	P value	95%CI	
intrcpt	-1.5821	2.4746	-0.6393	0.5226	-6.4323	3.268
Age_treatment	0.01	0.0372	0.2679	0.7888	-0.063	0.0829

Mixed-effects model (k = 27; τ^2 = 0.0834 [estimated amount of residual heterogeneity]; I^2 = 12.71% [residual heterogeneity/unaccounted variability]; R^2 = 0% [amount of heterogeneity accounted for]; p = 0.46, test for residual heterogeneity; p = 0.7888, test for moderators.

D. Gender with male proportion

1. Extracted data

Number	Study_ID	event.e	n.e	event.c	n.c	Gender_ treatment	Gender_ control	Gender_ difference
1	Abuzaiter 2023	3	80	0	85	0.41	0.34	0.07
2	Aljuhani 2021	0	49	1	49	0.27	0.06	0.20
3	Assor 2010	0	62	3	73	0.26	0.23	0.03
4	Buchalter 2021	71	14317	32	3982	0.52	0.47	0.05
5	Buchalter 2021 (2)	31	7046	22	2182	0.39	0.55	-0.16
6	Chin 2018	0	11	0	11	0.64	0.55	0.09
7	Chiu 2001	0	41	5	37	0.68	0.67	0.01
8	Chiu 2002	0	178	5	162	0.70	0.60	0.10
9	Cohen 2019	2	309	4	246	0.48	0.44	0.04
10	Crawford 2018	1	1070	7	815	0.49	0.48	0.01
11	Dial 2018	1	137	7	128	0.47	0.50	-0.03
12	Erken 2020	2	35	4	58	0.48	0.52	-0.03
13	Hanada 2019	5	110	7	92	0.25	0.24	0.01
14	Harper 2020	0	100	0	100	0.47	0.40	0.07
15	Khatri 2017	4	51	6	64	0.63	0.69	-0.06
16	Klasan 2021	0	301	0	331	0.42	0.43	-0.01
17	Koutalos 2020	2	142	2	178	0.29	0.28	0.01
18	Matziolis 2020	4	1082	92	7863	0.63	0.67	-0.04
19	McQueen 1990	2	204	2	201	0.42	0.33	0.08
20	Mulpur 2024	1	507	3	515	0.29	0.30	-0.01
21	Park 2021	0	488	3	572	0.41	0.42	-0.01
22	Parkinson 2021	1	725	16	1181	0.48	0.51	-0.03
23	Patel 2018	1	348	3	112	0.40	0.43	-0.03
24	Tahmasebi 2021	7	1710	6	314	0.19	0.20	-0.01
25	Wang 2023	0	45	6	45	0.53	0.51	0.02
26	Wininger 2024	0	10	0	10	0.30	0.50	-0.20
27	Wu 2022	0	45	4	45	0.53	0.51	0.02
28	Xu 2020	0	437	5	418	0.28	0.31	-0.03
29	Yavuz 2020	4	474	5	502	0.31	0.31	0.01
30	Zhengyuan 2024	0	60	0	60	0.15	0.17	-0.02

2. Meta-regression

(1) Gender_control+Gender_difference

Moderators	Estimate	SE	Z value	P value	95%CI	
intrcpt	-0.751	0.5373	-1.3976	0.1622	-1.8042	0.3022
Gender_control	-0.1787	1.1662	-0.1532	0.8782	-2.4644	2.107
Gender_difference	0.7421	2.1859	0.3395	0.7343	-3.5423	5.0264

Mixed-effects model (k = 30; τ^2 = 0.0673 [estimated amount of residual heterogeneity]; I^2 = 8.36% [residual heterogeneity/unaccounted variability]; R^2 = 0% [amount of heterogeneity accounted for]; p = 0.4925, test for residual heterogeneity; p = 0.8978, test for moderators.

(2) Gender_difference

Moderators	Estimate	SE	Z value	P value	95%CI	
intrcpt	-0.8041	0.1477	-5.4423	<0.0001*	-1.0936	-0.5145
Gender_difference	0.9847	1.8174	0.5418	0.588	-2.5774	4.5467

Mixed-effects model (k = 30; τ^2 = 0.0377 [estimated amount of residual heterogeneity]; I^2 = 5.13% [residual heterogeneity/unaccounted variability]; R^2 = 0% [amount of heterogeneity accounted for]; p = 0.5467, test for residual heterogeneity; p = 0.588, test for moderators.

*P<0.05, with statistical significance.

(3) Gender_treatment

Moderators	Estimate	SE	Z value	P value	95%CI	
intrcpt	-0.8307	0.5081	-1.635	0.1021	-1.8265	0.1651
Gender_treatment	-0.014	1.1093	0.0126	0.9899	-2.1602	2.1881

Mixed-effects model (k = 30; τ^2 = 0.0406 [estimated amount of residual heterogeneity]; I^2 = 6.5% [residual heterogeneity/unaccounted variability]; R^2 = 0% [amount of heterogeneity accounted for]; p = 0.5152, test for residual heterogeneity; p = 0.9899, test for moderators.

E. Diagnosis of diabetes mellitus proportion

1. Extracted data

Number	Study_ID	event.e	n.e	event.c	n.c	DM_ treatment	DM_ control	DM_ difference
1	Abuzaiter 2023	3	80	0	85	0.20	0.15	0.05
2	Buchalter 2021	71	14317	32	3982	0.08	0.22	-0.13
3	Buchalter 2021 (2)	31	7046	22	2182	0.07	0.23	-0.16
4	Chiu 2001	0	41	5	37	1.00	1.00	0.00
5	Crawford 2018	1	1070	7	815	0.14	0.16	-0.02
6	Dial 2018	1	137	7	128	0.12	0.15	-0.03
7	Hanada 2019	5	110	7	92	0.20	0.19	0.02
8	Harper 2020	0	100	0	100	0.26	0.20	0.06
9	Khatri 2017	4	51	6	64	0.33	0.36	-0.03
10	Klasan 2021	0	301	0	331	0.13	0.10	0.02
11	Mulpur 2024	1	507	3	515	0.34	0.31	0.02
12	Park 2021	0	488	3	572	0.23	0.23	0.00
13	Parkinson 2021	1	725	16	1181	0.09	0.14	-0.05
14	Patel 2018	1	348	3	112	0.10	0.13	-0.03
15	Xu 2020	0	437	5	418	0.10	0.14	-0.04
16	Yavuz 2020	4	474	5	502	0.22	0.26	-0.04

2. Meta-regression

(1) DM_control+DM_difference

Moderators	Estimate	SE	Z value	P value	95%CI	
intrcpt	-0.7293	0.5103	-1.4292	0.153	-1.7295	0.2709
DM_control	-0.3243	1.6777	-0.1933	0.8467	-3.6126	2.964
DM_difference	-0.6029	2.5732	-0.2343	0.8148	-5.6463	4.4405

Mixed-effects model (k = 16; τ^2 = 0.0461 [estimated amount of residual heterogeneity]; I^2 = 9.32% [residual heterogeneity/unaccounted variability]; R^2 = 0% [amount of heterogeneity accounted for]; p = 0.1847, test for residual heterogeneity; p = 0.9492, test for moderators.

(2) DM_difference

Moderators	Estimate	SE	Z value	P value	95%CI	
intrcpt	-0.8026	0.2888	-2.7793	0.0054*	-1.3686	-0.2366
DM_difference	-0.6839	2.4827	-0.2755	0.7829	-5.55	4.1821

Mixed-effects model (k = 16; τ^2 = 0.0388 [estimated amount of residual heterogeneity]; I^2 = 6.72% [residual heterogeneity/unaccounted variability]; R^2 = 0% [amount of heterogeneity accounted for]; p = 0.2372, test for residual heterogeneity; p = 0.7829, test for moderators.

*P<0.05, with statistical significance.

(3) DM_treatment

Moderators	Estimate	SE	Z value	P value	95%CI	
intrcpt	-0.6597	0.2278	-2.8962	0.0038*	-1.1061	-0.2132
DM_treatment	-0.4340	1.2782	-0.3395	0.7342	-2.9392	2.0713

Mixed-effects model (k = 16; τ^2 = 0.0151 [estimated amount of residual heterogeneity]; I^2 = 3.42% [residual heterogeneity/unaccounted variability]; R^2 = 0% [amount of heterogeneity accounted for]; p = 0.2382, test for residual heterogeneity; p = 0.7342, test for moderators.

*P<0.05, with statistical significance.

F. BMI

1. Extracted data

**Number**	**Study_ID**	**event.e**	**n.e**	**event.c**	**n.c**	**BMI_ treatment**	**BMI_ control**	**BMI_ difference**
1	Abuzaiter 2023	3	80	0	85	33.4	35.7	-2.3
2	Buchalter 2021	71	14317	32	3982	32.9	34.1	-1.2
3	Buchalter 2021 (2)	31	7046	22	2182	34.02	35.06	-1.04
4	Chin 2018	0	11	0	11	41	40	1
5	Crawford 2018	1	1070	7	815	31	31.1	-0.1
6	Dial 2018	1	137	7	128	30	29.8	0.2
7	Hanada 2019	5	110	7	92	26.7	25.7	1
8	Harper 2020	0	100	0	100	32	32	0
9	Klasan 2021	0	301	0	331	31.8	31.4	0.4
10	Matziolis 2020	4	1082	92	7863	29.5	29.8	-0.3
11	Mulpur 2024	1	507	3	515	28.5	28.4	0.1
12	Park 2021	0	488	3	572	31.99	32.47	-0.48
13	Parkinson 2021	1	725	16	1181	31.5	31.3	0.2
14	Patel 2018	1	348	3	112	30.6	31.1	-0.5
15	Wininger 2024	0	10	0	10	29.38	30.97	-1.59
16	Xu 2020	0	437	5	418	25.3	24.9	0.4
17	Yavuz 2020	4	474	5	502	29	28.9	0.1
18	Zhengyuan 2024	0	60	0	60	25.8	26.2	-0.4

2. Meta-regression

(1) BMI_control+BMI_difference

Moderators	Estimate	SE	Z value	P value	95%CI	
intrcpt	0.31	2.8537	0.1086	0.9135	-5.2831	5.903
BMI_control	-0.0418	0.0946	-0.4423	0.6583	-0.2273	0.1436
BMI_difference	-0.44	0.4003	-1.0992	0.2717	-1.2246	0.3446

Mixed-effects model (k = 18; τ^2 = 0 [estimated amount of residual heterogeneity]; I^2 = 0% [residual heterogeneity/unaccounted variability]; R^2 = 100% [amount of heterogeneity accounted for]; p = 0.5072, test for residual heterogeneity; p = 0.3443, test for moderators.

(2) BMI_difference

Moderators	Estimate	SE	Z value	P value	95%CI	
intrcpt	-0.9487	0.2115	-4.4856	<0.0001*	-1.3632	-0.5342
BMI_difference	-0.2886	0.2073	-1.3918	0.164	-0.6949	0.1178

Mixed-effects model (k = 18; τ^2 = 0 [estimated amount of residual heterogeneity]; I^2 = 0% [residual heterogeneity/unaccounted variability]; R^2 = 100% [amount of heterogeneity accounted for]; p = 0.5661, test for residual heterogeneity; p = 0.164, test for moderators.

*P<0.05, with statistical significance.

(3) BMI_treatment

Moderators	Estimate	SE	Z value	P value	95%CI	
intrcpt	-2.2758	2.0603	-1.1046	0.2693	-6.3139	1.7623
BMI_treatment	0.0474	0.0646	0.7348	0.4625	-0.0791	0.174

Mixed-effects model (k = 18; τ^2 = 0.0259 [estimated amount of residual heterogeneity]; I^2 = 4.98% [residual heterogeneity/unaccounted variability]; R^2 = 0% [amount of heterogeneity accounted for]; p = 0.4697, test for residual heterogeneity; p = 0.4625, test for moderators.

G. Diagnosis of rheumatic arthritis

1. Extracted data

Number	Study_ID	event.e	n.e	event.c	n.c	RA_ treatment	RA_ control	RA_ difference
1	Buchalter 2021	71	14317	32	3982	0.07	0.03	0.04
2	Buchalter 2021 (2)	31	7046	22	2182	0.13	0.00	0.13
3	Crawford 2018	1	1070	7	815	0.06	0.03	0.04
4	Dial 2018	1	137	7	128	0.07	0.06	0.01
5	Harper 2020	0	100	0	100	0.02	0.03	-0.01
6	Park 2021	0	488	3	572	0.06	0.06	0.00

2. Meta-regression

(1) RA_control+RA_difference

Moderators	Estimate	SE	Z value	P value	95%CI	
intrcpt	0.7366	2.9473	-0.2499	0.8026	-5.04	6.5132
RA_control	-41.2535	61.1409	-0.6747	0.4998	-161.0874	78.5805
RA_difference	-11.8583	23.9568	-0.495	0.6206	-58.8128	35.0962

Mixed-effects model (k = 6; τ^2 = 0.3858 [estimated amount of residual heterogeneity]; I^2 = 38.89% [residual heterogeneity/unaccounted variability]; R^2 = 0% [amount of heterogeneity accounted for]; p = 0.2266, test for residual heterogeneity; p = 0.7286, test for moderators.

(2) RA_difference

Moderators	Estimate	SE	Z value	P value	95%CI	
intrcpt	-1.1923	0.6279	-1.8989	0.0576	-2.423	0.0384
RA_difference	-3.2873	7.8593	-0.4183	0.6757	-12.1166	18.6913

Mixed-effects model (k = 6; τ^2 = 0.364 [estimated amount of residual heterogeneity]; I^2 = 34.73% [residual heterogeneity/unaccounted variability]; R^2 = 0% [amount of heterogeneity accounted for]; p = 0.2605, test for residual heterogeneity; p = 0.6757, test for moderators.

(3) RA_treatment

Moderators	Estimate	SE	Z value	P value	95%CI	
intrcpt	-1.2613	1.1129	-1.1334	0.2571	-3.4425	0.9199
RA_treatment	3.2509	12.0253	0.2703	0.7869	-20.3182	26.82

Mixed-effects model (k = 6; τ^2 = 0.3473 [estimated amount of residual heterogeneity]; I^2 = 33% [residual heterogeneity/unaccounted variability]; R^2 = 0% [amount of heterogeneity accounted for]; p = 0.2695, test for residual heterogeneity; p = 0.7869, test for moderators.

H. Smoking

1. Extracted data

Number	Study_ID	event.e	n.e	event.c	n.c	Smoking_ treatment	Smoking_ control	Smoking_ difference
1	Abuzaiter 2023	3	80	0	85	0.11	0.05	0.07
2	Dial 2018	1	137	7	128	0.16	0.15	0.01
3	Khatri 2017	4	51	6	64	0.12	0.08	0.04
4	Mulpur 2024	1	507	3	515	0.11	0.12	0.00
5	Park 2021	0	488	3	572	0.05	0.05	0.00
6	Parkinson 2021	1	725	16	1181	0.04	0.02	0.02
7	Patel 2018	1	348	3	112	0.11	0.13	-0.02
8	Xu 2020	0	437	5	418	0.22	0.21	0.01

2. Meta-regression

(1) Smoking_control+Smoking_difference

Moderators	Estimate	SE	Z value	P value	95%CI	
intrcpt	-2.0481	1.0782	-1.8995	0.0575	-4.1614	0.0652
Smoking_control	-0.0345	8.3745	-0.0041	0.9967	-16.4482	16.3792
Smoking _difference	44.6953	18.6953	2.3907	0.0168*	-8.0531	81.3375

Mixed-effects model (k = 8; τ^2 = 0 [estimated amount of residual heterogeneity]; I^2 = 0% [residual heterogeneity/unaccounted variability]; R^2 = 100% [amount of heterogeneity accounted for]; p = 0.6081, test for residual heterogeneity; p = 0.0308, test for moderators.

*P<0.05, with statistical significance.

(2) Smoking_difference

Moderators	Estimate	SE	Z value	P value	95%CI	
intrcpt	-2.052	0.5206	-3.9417	<0.0001*	-3.0724	-1.0317
Smoking_difference	44.7277	16.9566	2.6378	0.0083*	11.4934	77.962

Mixed-effects model (k = 8; τ^2 = 0 [estimated amount of residual heterogeneity]; I^2 = 0% [residual heterogeneity/unaccounted variability]; R^2 = 100% [amount of heterogeneity accounted for]; p = 0.7304, test for residual heterogeneity; p = 0.0083, test for moderators.

*P<0.05, with statistical significance.

(3) Smoking_treatment

Moderators	Estimate	SE	Z value	P value	95%CI	
intrcpt	-1.1694	1.2815	-0.9126	0.3615	-3.681	1.3421
Smoking_treatment	-0.5563	10.2968	-0.054	0.9569	-20.7376	19.625

Mixed-effects model (k = 8; τ^2 = 0.7701 [estimated amount of residual heterogeneity]; I^2 = 40% [residual heterogeneity/unaccounted variability]; R^2 = 0% [amount of heterogeneity accounted for]; p = 0.1032, test for residual heterogeneity; p = 0.9569, test for moderators.
