# Supplementary material for: Effectiveness of Local Antibiotics for Infection Prevention in Primary Joint Arthroplasty: A Systematic Review and Meta-Analysis
Source: Antibiotics (Basel). 2025 Feb 20;14(3):214. doi: 10.3390/antibiotics14030214 (PMC11939600; doi:10.3390/antibiotics14030214)
Supplement: Supplementary file 1 [file antibiotics-14-00214-s001.zip › Supplementary Material File S4.pdf]

Supplementary Material File S4 (Meta-regression of Powder Data)

A. Study design

1. Extracted data

Number	Study_ID	event.e	n.e	event.c	n.c	Design
1	Abuzaiter 2023	3	80	0	85	RCT
2	Aljuhani 2021	0	49	1	49	Cohort
3	Assor 2010	0	62	3	73	Cohort
4	Buchalter 2021	71	14317	32	3982	Cohort
5	Buchalter 2021 (2)	31	7046	22	2182	Cohort
6	Cohen 2019	2	309	4	246	Cohort
7	Crawford 2018	1	1070	7	815	Cohort
8	Dial 2018	1	137	7	128	Cohort
9	Erken 2020	2	35	4	58	Cohort
10	Hanada 2019	5	110	7	92	Cohort
11	Khatri 2017	4	51	6	64	Cohort
12	Koutalos 2020	2	142	2	178	Cohort
13	Matziolis 2020	4	1082	92	7863	Cohort
14	Mulpur 2024	1	507	3	515	RCT
15	Patel 2018	1	348	3	112	Cohort
16	Tahmasebi 2021	7	1710	6	314	Cohort
17	Wang 2023	0	45	6	45	RCT
18	Wu 2022	0	45	4	45	RCT
19	Xu 2020	0	437	5	418	Cohort
20	Yavuz 2020	4	474	5	502	Cohort
21	Zhengyuan 2024	0	60	0	60	RCT

2. Meta-regression

Moderators	Estimate	SE	Z value	P value	95%CI	
intrcpt	-0.7568	0.1432	-5.2843	<0.0001*	-1.0375	-0.4761
DesignRCT	-0.1467	0.6667	-0.2201	0.8258	-1.4534	1.1599

Mixed-effects model (k = 21; τ^2 = 0.0165 [estimated amount of residual heterogeneity]; I^2 = 3.94% [residual heterogeneity/unaccounted variability]; R^2 = 0% [amount of heterogeneity accounted for]; p = 0.3698, test for residual heterogeneity; p = 0.8258, test for moderators.

*P<0.05, with statistical significance.

B. Age

1. Extracted data

Number	Study_ID	event.e	n.e	event.c	n.c	Age_ treatment	Age_ control	Age_ difference
1	Abuzaiter 2023	3	80	0	85	66	64	2
2	Assor 2010	0	62	3	73	73	72	1
3	Buchalter 2021	71	14317	32	3982	62.97	63.34	-0.37
4	Buchalter 2021 (2)	31	7046	22	2182	63.74	63.82	-0.08
5	Cohen 2019	2	309	4	246	66	67.3	-1.3
6	Crawford 2018	1	1070	7	815	64.8	63.3	1.5
7	Dial 2018	1	137	7	128	61.2	61.5	-0.3
8	Erken 2020	2	35	4	58	81.88	81.87	0.01
9	Hanada 2019	5	110	7	92	74.6	73.3	1.3
10	Matziolis 2020	4	1082	92	7863	69	68	1
11	Mulpur 2024	1	507	3	515	61.7	61.4	0.3
12	Patel 2018	1	348	3	112	63.6	64.9	-1.3
13	Tahmasebi 2021	7	1710	6	314	65	66.4	-1.4
14	Wang 2023	0	45	6	45	67.9	68	-0.1
15	Wu 2022	0	45	4	45	67.9	68	-0.1
16	Xu 2020	0	437	5	418	66.9	67.1	-0.2
17	Yavuz 2020	4	474	5	502	65.5	63.4	2.1
18	Zhengyuan 2024	0	60	0	60	68.3	66.5	1.8

2. Meta-regression

(1) Age_control+Age_difference

Moderators	Estimate	SE	Z value	P value	95%CI	
intrcpt	-1.2513	2.781	-0.45	0.6527	-6.702	4.1993
Age_control	0.0048	0.042	0.1137	0.9095	-0.0775	0.087
Age_difference	0.2515	0.1929	1.3036	0.1924	-0.1266	0.6297

Mixed-effects model (k = 18; τ^2 = 0.1146 [estimated amount of residual heterogeneity]; I^2 = 21.69% [residual heterogeneity/unaccounted variability]; R^2 = 0% [amount of heterogeneity accounted for]; p = 0.2983, test for residual heterogeneity; p = 0.4067, test for moderators.

(2) Age_difference

Moderators	Estimate	SE	Z value	P value	95%CI	
intrcpt	-0.9135	0.18	-5.0748	<0.0001*	-1.2663	-0.5607
Age_difference	0.2435	0.1854	1.3132	0.1891	-0.1199	0.607

Mixed-effects model (k = 18; τ^2 = 0.088 [estimated amount of residual heterogeneity]; I^2 = 17.93% [residual heterogeneity/unaccounted variability]; R^2 = 0% [amount of heterogeneity accounted for]; p = 0.3547, test for residual heterogeneity; p = 0.1891, test for moderators.

*P<0.05, with statistical significance.

(3) Age_treatment

Moderators	Estimate	SE	Z value	P value	95%CI	
intrcpt	-2.1849	2.5165	-0.8682	0.3853	-7.1172	2.7474
Age_treatment	0.0197	0.0379	0.5179	0.6045	-0.0547	0.094

Mixed-effects model (k = 18; τ^2 = 0.0849 [estimated amount of residual heterogeneity]; I^2 = 17.05% [residual heterogeneity/unaccounted variability]; R^2 = 0% [amount of heterogeneity accounted for]; p = 0.2923, test for residual heterogeneity; p = 0.6045, test for moderators.

C. Gender with male proportion

1. Extracted data

Number	Study_ID	event.e	n.e	event.c	n.c	Gender_ treatment	Gender_ control	Gender_ difference
1	Abuzaiter 2023	3	80	0	85	0.41	0.34	0.07
2	Aljuhani 2021	0	49	1	49	0.27	0.06	0.20
3	Assor 2010	0	62	3	73	0.26	0.23	0.03
4	Buchalter 2021	71	14317	32	3982	0.52	0.47	0.05
5	Buchalter 2021 (2)	31	7046	22	2182	0.39	0.55	-0.16
6	Cohen 2019	2	309	4	246	0.48	0.44	0.04
7	Crawford 2018	1	1070	7	815	0.49	0.48	0.01
8	Dial 2018	1	137	7	128	0.47	0.50	-0.03
9	Erken 2020	2	35	4	58	0.48	0.52	-0.03
10	Hanada 2019	5	110	7	92	0.25	0.24	0.01
11	Khatri 2017	4	51	6	64	0.63	0.69	-0.06
12	Koutalos 2020	2	142	2	178	0.29	0.28	0.01
13	Matziolis 2020	4	1082	92	7863	0.63	0.67	-0.04
14	Mulpur 2024	1	507	3	515	0.29	0.30	-0.01
15	Patel 2018	1	348	3	112	0.40	0.43	-0.03
16	Tahmasebi 2021	7	1710	6	314	0.19	0.20	-0.01
17	Wang 2023	0	45	6	45	0.53	0.51	0.02
18	Wu 2022	0	45	4	45	0.53	0.51	0.02
19	Xu 2020	0	437	5	418	0.28	0.31	-0.03
20	Yavuz 2020	4	474	5	502	0.31	0.31	0.01
21	Zhengyuan 2024	0	60	0	60	0.15	0.17	-0.02

2. Meta-regression

(1) Gender_control+Gender_difference

Moderators	Estimate	SE	Z value	P value	95%CI	
intrcpt	-0.999	0.5245	-1.9047	0.0568	-2.0269	0.029
Gender_control	-0.5811	1.1355	-0.5118	0.6088	-1.6444	2.8067
Gender_difference	1.6766	1.9507	0.8595	0.3901	-2.1467	5.4999

Mixed-effects model (k = 21; τ^2 = 0.0202 [estimated amount of residual heterogeneity]; I^2 = 3.25% [residual heterogeneity/unaccounted variability]; R^2 = 0% [amount of heterogeneity accounted for]; p = 0.37, test for residual heterogeneity; p = 0.6818, test for moderators.

(2) Gender_difference

Moderators	Estimate	SE	Z value	P value	95%CI	
intrcpt	-0.7391	0.1457	-5.0723	<0.0001*	-1.0247	-0.4535
Gender_difference	1.2657	1.7516	0.7226	0.4699	-2.1674	4.6988

Mixed-effects model (k = 21; τ^2 = 0.0184[estimated amount of residual heterogeneity]; I^2 = 3.18% [residual heterogeneity/unaccounted variability]; R^2 = 0% [amount of heterogeneity accounted for]; p = 0.4126, test for residual heterogeneity; p = 0.4699, test for moderators.

*P<0.05, with statistical significance.

(3) Gender_treatment

Moderators	Estimate	SE	Z value	P value	95%CI	
intrcpt	-1.1087	0.4886	-2.269	0.0233*	-2.0665	-0.151
Gender_treatment	0.8296	1.0605	0.7823	0.434	-1.2488	2.9081

Mixed-effects model (k = 21; τ^2 = 0 [estimated amount of residual heterogeneity]; I^2 = 0% [residual heterogeneity/unaccounted variability]; R^2 = 100% [amount of heterogeneity accounted for]; p = 0.4022, test for residual heterogeneity; p = 0.434, test for moderators.

D. Diagnosis of diabetes mellitus proportion

1. Extracted data

Number	Study_ID	event.e	n.e	event.c	n.c	DM_ treatment	DM_ control	DM_ difference
1	Abuzaiter 2023	3	80	0	85	0.20	0.15	0.05
2	Buchalter 2021	71	14317	32	3982	0.08	0.22	-0.13
3	Buchalter 2021 (2)	31	7046	22	2182	0.07	0.23	-0.16
4	Crawford 2018	1	1070	7	815	0.14	0.16	-0.02
5	Dial 2018	1	137	7	128	0.12	0.15	-0.03
6	Hanada 2019	5	110	7	92	0.20	0.19	0.02
7	Khatri 2017	4	51	6	64	0.33	0.36	-0.03
8	Mulpur 2024	1	507	3	515	0.34	0.31	0.02
9	Patel 2018	1	348	3	112	0.10	0.13	-0.03
10	Xu 2020	0	437	5	418	0.10	0.14	-0.04
11	Yavuz 2020	4	474	5	502	0.22	0.26	-0.04

2. Meta-regression

(1) DM_control+DM_difference

Moderators	Estimate	SE	Z value	P value	95%CI	
intrcpt	-1.809	0.8653	-2.0905	0.0366*	-3.505	-0.113
DM_control	-5.1406	3.539	1.4525	0.1464	-1.7958	12.077
DM_difference	0.0817	2.5716	0.0318	0.9747	-4.9586	5.122

Mixed-effects model (k = 11; τ^2 = 0.0206 [estimated amount of residual heterogeneity]; I^2 = 6.05% [residual heterogeneity/unaccounted variability]; R^2 = 0% [amount of heterogeneity accounted for]; p = 0.2283, test for residual heterogeneity; p = 0.347, test for moderators.

*P<0.05, with statistical significance.

(2) DM_difference

Moderators	Estimate	SE	Z value	P value	95%CI	
intrcpt	-06232	0.3078	-2.0246	0.0429*	-1.2265	-0.0199
DM_difference	0.2077	2.4576	0.0845	0.9326	-4.6092	5.0246

Mixed-effects model (k = 11; τ^2 = 0.0038 [estimated amount of residual heterogeneity]; I^2 = 1.13% [residual heterogeneity/unaccounted variability]; R^2 = 0% [amount of heterogeneity accounted for]; p = 0.1802, test for residual heterogeneity; p = 0.9326, test for moderators.

*P<0.05, with statistical significance.

(3) DM_treatment

Moderators	Estimate	SE	Z value	P value	95%CI	
intrcpt	-0.8651	0.2836	-3.0505	0.0023*	-1.4209	-0.3093
DM_treatment	1.7969	2.0034	0.8969	0.3698	-2.1296	5.7234

Mixed-effects model (k = 11; τ^2 = 0.0115 [estimated amount of residual heterogeneity]; I^2 = 3.45% [residual heterogeneity/unaccounted variability]; R^2 = 0% [amount of heterogeneity accounted for]; p = 0.2213, test for residual heterogeneity; p = 0.3698, test for moderators.

*P<0.05, with statistical significance.

E. BMI

1. Extracted data

Number	Study_ID	event.e	n.e	event.c	n.c	BMI_ treatment	BMI_ control	BMI_ difference
1	Abuzaiter 2023	3	80	0	85	33.4	35.7	-2.3
2	Buchalter 2021	71	14317	32	3982	32.9	34.1	-1.2
3	Buchalter 2021 (2)	31	7046	22	2182	34.02	35.06	-1.04
4	Crawford 2018	1	1070	7	815	31	31.1	-0.1
5	Dial 2018	1	137	7	128	30	29.8	0.2
6	Hanada 2019	5	110	7	92	26.7	25.7	1
7	Matziolis 2020	4	1082	92	7863	29.5	29.8	-0.3
8	Mulpur 2024	1	507	3	515	28.5	28.4	0.1
9	Patel 2018	1	348	3	112	30.6	31.1	-0.5
10	Xu 2020	0	437	5	418	25.3	24.9	0.4
11	Yavuz 2020	4	474	5	502	29	28.9	0.1
12	Zhengyuan 2024	0	60	0	60	25.8	26.2	-0.4

2. Meta-regression

(1) BMI_control+BMI_difference

Moderators	Estimate	SE	Z value	P value	95%CI	
intrcpt	3.5451	4.4074	0.8044	0.4212	-5.0932	12.1834
BMI_control	-0.1512	0.1489	-1.0151	0.3101	-0.443	0.1407
BMI_difference	-0.8964	0.6522	-1.3745	0.1693	-2.1748	0.3819

Mixed-effects model (k = 12; τ^2 = 0 [estimated amount of residual heterogeneity]; I^2 = 0% [residual heterogeneity/unaccounted variability]; R^2 = 99.97% [amount of heterogeneity accounted for]; p = 0.3183, test for residual heterogeneity; p = 0.2753, test for moderators.

(2) BMI_difference

Moderators	Estimate	SE	Z value	P value	95%CI	
intrcpt	-0.9227	0.2267	-4.0702	<0.0001*	-1.3671	-0.4784
BMI_difference	-0.2729	0.2192	-1.2449	0.2132	-0.7026	0.1568

Mixed-effects model (k = 12; τ^2 = 0 [estimated amount of residual heterogeneity]; I^2 = 0% [residual heterogeneity/unaccounted variability]; R^2 = 100% [amount of heterogeneity accounted for]; p = 0.3242, test for residual heterogeneity; p = 0.2132, test for moderators.

*P<0.05, with statistical significance.

(3) BMI_treatment

Moderators	Estimate	SE	Z value	P value	95%CI	
intrcpt	-2.1555	2.1515	-1.0018	0.3164	-6.3724	2.0615
BMI_treatment	0.0447	0.0674	0.6622	0.5078	-0.0875	0.1769

Mixed-effects model (k = 12; τ^2 = 0.0227 [estimated amount of residual heterogeneity]; I^2 = 6.13% [residual heterogeneity/unaccounted variability]; R^2 = 0% [amount of heterogeneity accounted for]; p = 0.2524, test for residual heterogeneity; p = 0.5078, test for moderators.

F. Diagnosis of rheumatic arthritis

1. Extracted data

Number	Study_ID	event.e	n.e	event.c	n.c	RA_ treatment	RA_ control	RA_ difference
1	Buchalter 2021	71	14317	32	3982	0.07	0.03	0.04
2	Buchalter 2021 (2)	31	7046	22	2182	0.13	0.00	0.13
3	Crawford 2018	1	1070	7	815	0.06	0.03	0.04
4	Dial 2018	1	137	7	128	0.07	0.06	0.01

2. Meta-regression

(1) RA_control+RA_difference

Moderators	Estimate	SE	Z value	P value	95%CI	
intrcpt	-1.1326	5.523	-0.2051	0.8375	-11.9576	9.6923
RA_control	-9.1007	110.1761	-0.0826	0.9342	-225.0418	206.8404
RA_difference	2.9996	45.5329	0.0659	0.9475	-86.2431	92.2424

Mixed-effects model (k = 4; $\tau^2 = 1.2149$ [estimated amount of residual heterogeneity]; $I^2 = 75.61\%$ [residual heterogeneity/unaccounted variability]; $R^2 = 0\%$ [amount of heterogeneity accounted for]; p = 0.0429, test for residual heterogeneity; p = 0.8891, test for moderators.

(2) RA_difference

Moderators	Estimate	SE	Z value	P value	95%CI	
intrcpt	-1.4124	0.8647	-1.6334	0.1024	-3.1071	0.2824
RA_difference	5.2375	10.6383	0.4923	0.6225	-15.6132	26.0882

Mixed-effects model (k = 4; $\tau^2 = 0.6341$[estimated amount of residual heterogeneity]; $I^2 = 55.57\%$ [residual heterogeneity/unaccounted variability]; $R^2 = 0\%$ [amount of heterogeneity accounted for]; p = 0.1031, test for residual heterogeneity; p = 0.6225, test for moderators.

(3) RA_treatment

Moderators	Estimate	SE	Z value	P value	95%CI	
intrcpt	-1.7825	1.6346	-1.0905	0.2755	-4.9862	1.4212
RA_treatment	7.9509	17.4304	0.4562	0.6483	-26.2121	42.1138

Mixed-effects model (k = 4; $\tau^2 = 0.6704$ [estimated amount of residual heterogeneity]; $I^2 = 55.02\%$ [residual heterogeneity/unaccounted variability]; $R^2 = 0\%$ [amount of heterogeneity accounted for]; p = 0.106, test for residual heterogeneity; p = 0.6483, test for moderators.

G. Smoking

1. Extracted data

Number	Study_ID	event.e	n.e	event.c	n.c	Smoking_ treatment	Smoking_ control	Smoking_ difference
1	Abuzaiter 2023	3	80	0	85	0.11	0.05	0.07
2	Dial 2018	1	137	7	128	0.16	0.15	0.01
3	Khatri 2017	4	51	6	64	0.12	0.08	0.04
4	Mulpur 2024	1	507	3	515	0.11	0.12	0.00
5	Patel 2018	1	348	3	112	0.11	0.13	-0.02
6	Xu 2020	0	437	5	418	0.22	0.21	0.01

2. Meta-regression

(1) Smoking_control+Smoking_difference

Moderators	Estimate	SE	Z value	P value	95%CI	
intrcpt	0.3027	1.8821	0.1608	0.8722	-3.3862	3.9915
Smoking_control	-16.1313	13.2942	-1.2134	0.225	-42.1873	9.9248
Smoking _difference	25.0582	23.4684	1.0677	0.2856	-20.9391	71.0555

Mixed-effects model (k = 6; τ^2 = 0 [estimated amount of residual heterogeneity]; I^2 = 0% [residual heterogeneity/unaccounted variability]; R^2 = 100% [amount of heterogeneity accounted for]; p = 0.8609, test for residual heterogeneity; p = 0.0194, test for moderators.

(2) Smoking_difference

Moderators	Estimate	SE	Z value	P value	95%CI	
intrcpt	-1.8709	0.5775	-3.2398	0.0012*	-3.0028	-0.7391
Smoking_difference	44.1352	17.4239	2.533	0.0113*	9.985	78.2855

Mixed-effects model (k = 6; τ^2 = 0 [estimated amount of residual heterogeneity]; I^2 = 0% [residual heterogeneity/unaccounted variability]; R^2 = 100% [amount of heterogeneity accounted for]; p = 0.6946, test for residual heterogeneity; p = 0.0113, test for moderators.

*P<0.05, with statistical significance.

(3) Smoking_treatment

Moderators	Estimate	SE	Z value	P value	95%CI	
intrcpt	1.6632	2.0199	0.8234	0.4103	-2.2957	5.6221
Smoking_treatment	-19.5866	14.6872	-1.3336	0.1823	-48.3731	9.1998

Mixed-effects model (k = 6; τ^2 = 0.3702 [estimated amount of residual heterogeneity]; I^2 = 25.32% [residual heterogeneity/unaccounted variability]; R^2 = 45.94% [amount of heterogeneity accounted for]; p = 0.1824, test for residual heterogeneity; p = 0.1823, test for moderators.
