# Supplementary material for: Effectiveness of Local Antibiotics for Infection Prevention in Primary Joint Arthroplasty: A Systematic Review and Meta-Analysis
Source: Antibiotics (Basel). 2025 Feb 20;14(3):214. doi: 10.3390/antibiotics14030214 (PMC11939600; doi:10.3390/antibiotics14030214)
Supplement: Supplementary file 1 [file antibiotics-14-00214-s001.zip › Supplementary Material File S5.pdf]

Supplementary Material File S5 (Meta-regression of Powder Data without Abuzaiter 2023 [7])

A. Study design

1. Extracted data

Number	Study_ID	event.e	n.e	event.c	n.c	Design
1	Aljuhani 2021	0	49	1	49	Cohort
2	Assor 2010	0	62	3	73	Cohort
3	Buchalter 2021	71	14317	32	3982	Cohort
4	Buchalter 2021 (2)	31	7046	22	2182	Cohort
5	Cohen 2019	2	309	4	246	Cohort
6	Crawford 2018	1	1070	7	815	Cohort
7	Dial 2018	1	137	7	128	Cohort
8	Erken 2020	2	35	4	58	Cohort
9	Hanada 2019	5	110	7	92	Cohort
10	Khatri 2017	4	51	6	64	Cohort
11	Koutalos 2020	2	142	2	178	Cohort
12	Matziolis 2020	4	1082	92	7863	Cohort
13	Mulpur 2024	1	507	3	515	RCT
14	Patel 2018	1	348	3	112	Cohort
15	Tahmasebi 2021	7	1710	6	314	Cohort
16	Wang 2023	0	45	6	45	RCT
17	Wu 2022	0	45	4	45	RCT
18	Xu 2020	0	437	5	418	Cohort
19	Yavuz 2020	4	474	5	502	Cohort
20	Zhengyuan 2024	0	60	0	60	RCT

2. Meta-regression

Moderators	Estimate	SE	Z value	P value	95%CI	
intrcpt	-0.7513	0.1404	-5.3527	<0.0001*	-1.0264	-0.4762
DesignRCT	-0.8174	0.7344	-1.113	0.2657	-2.2568	0.622

Mixed-effects model (k = 20; τ^2 = 0.0121 [estimated amount of residual heterogeneity]; I^2 = 3.04% [residual heterogeneity/unaccounted variability]; R^2 = 46.05% [amount of heterogeneity accounted for]; p = 0.6052, test for residual heterogeneity; p = 0.2657, test for moderators.

*P<0.05, with statistical significance.
