# Supplementary material for: Effectiveness of Local Antibiotics for Infection Prevention in Primary Joint Arthroplasty: A Systematic Review and Meta-Analysis
Source: Antibiotics (Basel). 2025 Feb 20;14(3):214. doi: 10.3390/antibiotics14030214 (PMC11939600; doi:10.3390/antibiotics14030214)
Supplement: Supplementary file 1 [file antibiotics-14-00214-s001.zip › Supplementary Material File S6.pdf]

Supplementary Material File S6 (Meta-regression of Overall Data without Intraosseous and Abuzaiter 2023 [7])

A. Administration type

1. Extracted data

Number	Study_ID	event.e	n.e	event.c	n.c	Administration
1	Aljuhani 2021	0	49	1	49	powder
2	Assor 2010	0	62	3	73	powder
3	Buchalter 2021	71	14317	32	3982	powder
4	Buchalter 2021 (2)	31	7046	22	2182	powder
5	Chiu 2001	0	41	5	37	cement
6	Chiu 2002	0	178	5	162	cement
7	Cohen 2019	2	309	4	246	powder
8	Crawford 2018	1	1070	7	815	powder
9	Dial 2018	1	137	7	128	powder
10	Erken 2020	2	35	4	58	powder
11	Hanada 2019	5	110	7	92	powder
12	Hinarejos 2013	20	1483	20	1465	cement
13	Josefsson 1993	3	853	13	835	cement
14	Khatri 2017	4	51	6	64	powder
15	Koutalos 2020	2	142	2	178	powder
16	Matziolis 2020	4	1082	92	7863	powder
17	McQueen 1990	2	204	2	201	cement
18	Mulpur 2024	1	507	3	515	powder
19	Namba 2009	28	2030	154	20859	cement
20	Patel 2018	1	348	3	112	powder
21	Tahmasebi 2021	7	1710	6	314	powder
22	Wang 2023	0	45	6	45	powder
23	Wu 2022	0	45	4	45	powder
24	Xu 2020	0	437	5	418	powder
25	Yavuz 2020	4	474	5	502	powder
26	Zhengyuan 2024	0	60	0	60	powder

2. Meta-regression

Moderators	Estimate	SE	Z value	P value	95%CI	
intrcpt	-0.20042	0.3107	-0.6571	0.5111	-0.8132	0.4048
powder	-0.6997	0.371	-1.8859	0.0593	-1.4268	0.0275

Mixed-effects model (k = 26; τ^2 = 0.2153 [estimated amount of residual heterogeneity]; I^2 = 38.06% [residual heterogeneity/unaccounted variability]; R^2 = 40.09% [amount of heterogeneity accounted for]; p = 0.0591, test for residual heterogeneity; p = 0.0593, test for moderators.
